# The association of HDL-cholesterol levels with incident major adverse cardiovascular events and mortality in 0.6 million individuals with type 2 diabetes: a population-based retrospective cohort study

**DOI:** 10.1186/s12916-024-03810-4

**Published:** 2024-12-18

**Authors:** David Tak Wai Lui, Lanlan Li, Xiaodong Liu, Xi Xiong, Eric Ho Man Tang, Chi Ho Lee, Yu Cho Woo, Brian Hung Hin Lang, Carlos King Ho Wong, Kathryn Choon Beng Tan

**Affiliations:** 1 Department of Medicine, School of Clinical Medicine, Li Ka Shing Faculty of Medicine, The University of Hong Kong, Hong Kong SAR, China; 2 State Key Laboratory of Pharmaceutical Biotechnology, The University of Hong Kong, Hong Kong SAR, China; 3 Department of Surgery, School of Clinical Medicine, Li Ka Shing Faculty of Medicine, The University of Hong Kong, Hong Kong SAR, China; 4 Laboratory of Data Discovery for Health (D24H), Hong Kong SAR, China; 5 Department of Pharmacology and Pharmacy, Li Ka Shing Faculty of Medicine, The University of Hong Kong, Hong Kong SAR, China; 6 Research Department of Practice and Policy, School of Pharmacy, University College London, London, UK; 7 Department of Family Medicine and Primary Care, School of Clinical Medicine, Li Ka Shing Faculty of Medicine, The University of Hong Kong, Hong Kong SAR, China; 8 Department of Infectious Disease Epidemiology & Dynamics, Faculty of Epidemiology and Population Health, London School of Hygiene and Tropical Medicine, London, UK

**Keywords:** HDL cholesterol, Type 2 diabetes, Cardiovascular, Mortality, Dyslipidemia

## Abstract

**Background:**

High levels of high-density lipoprotein cholesterol (HDL-C) are previously considered protective against cardiovascular diseases (CVD), but recent studies suggest an increased risk of adverse events at very high HDL-C levels in the general population. It remains to be elucidated such a relationship in diabetes, a condition with high cardiovascular risks. We examined the association of HDL-C levels with the risk of major adverse cardiovascular events (MACE) and mortality in type 2 diabetes.

**Methods:**

This retrospective cohort study identified individuals with type 2 diabetes who had HDL-C records (2008–2020) from the electronic health record database of the Hong Kong Hospital Authority. They were classified into three groups based on their first-recorded HDL-C levels following diabetes diagnosis: low (≤ 40 mg/dL), medium (> 40 and ≤ 80 mg/dL) and high HDL-C (> 80 mg/dL) groups. The primary outcome was incident MACE (composite of myocardial infarction, stroke, heart failure, and cardiovascular mortality). Cox regression model and restricted cubic spline analysis were employed to assess the relationship between HDL-C and adverse outcomes.

**Results:**

Among 596,943 individuals with type 2 diabetes included, 168,931 (28.30%), 412,863 (69.16%), and 15,149 (2.54%) were classified as low HDL-C, medium HDL-C, and high HDL-C groups, respectively. Over a median follow-up of 79.5 months, both low and high HDL-C groups had higher risk of incident MACE compared to the medium HDL-C group (HR 1.24, 95% CI 1.23–1.26, *P* < 0.001; HR 1.09, 95% CI 1.04–1.13, *P* < 0.001). The spline curves revealed a U-shaped association between HDL-C levels and incident MACE (non-linear *p* < 0.001). Similar U-shaped relationship was observed for all-cause and non-cardiovascular mortality.

**Conclusions:**

Our study demonstrated a U-shaped association between HDL-C levels and incident MACEs and all-cause and non-cardiovascular mortality in individuals with type 2 diabetes, highlighting the need for mechanistic studies on the adverse outcomes seen at high HDL-C levels in type 2 diabetes.

**Supplementary Information:**

The online version contains supplementary material available at 10.1186/s12916-024-03810-4.

## Background

The Framingham Study [1] and others [2] have unequivocally established low high-density lipoprotein cholesterol (HDL-C) levels as a risk factor for atherosclerotic cardiovascular diseases. HDL-C levels have been reported to inversely correlate with the risk of cardiovascular disease (CVD), with each unit increase in HDL-C associated with decreased CVD risk by 2–3% [2–5]. However, clinical trials with niacin and cholesteryl ester transfer protein (CETP) inhibitors failed to reduce cardiovascular risk despite markedly increasing HDL-C levels [6, 7]. Mendelian randomization studies also suggested that genetic variations that increased HDL-C levels were not associated with lower cardiovascular risks [8]. Indeed, higher HDL-C levels are not always protective [9]. Recent studies have indicated a U-shaped relationship between HDL-C levels and CVD risks in the general population [10], in individuals with hypertension [11], and in individuals with coronary artery disease [12], indicating that extremely high HDL-C levels are associated with increased cardiovascular risks.

Type 2 diabetes is a global health problem with a prevalence of 10% [13], which is associated with significantly elevated cardiovascular risks [14]. Diabetic dyslipidaemia, characterized by low HDL-C and high triglyceride levels, is frequently seen in individuals with type 2 diabetes [15]. Several studies which have explored the relationships between HDL-C levels and cardiovascular outcomes in a sub-cohort of subjects with type 2 diabetes yielded inconsistent results [12, 16, 17], limited by the sample size of the sub-cohort. Furthermore, the influence of various diabetes-specific factors, such as glycaemic control and diabetic complications, on the relationships between HDL-C levels and cardiovascular outcomes remains to be evaluated. Since statin therapy effectively reduces low-density lipoprotein cholesterol (LDL-C) levels and the cardiovascular risks [18], whether statin therapy modulates the association between HDL-C levels and cardiovascular outcomes is less well known. To address these knowledge gaps regarding the role of HDL-C in the prediction of cardiovascular risks in individuals with type 2 diabetes, we carried out a study in a well-characterized population-based cohort of individuals with type 2 diabetes to clarify the association of HDL-C levels with adverse cardiovascular outcomes and mortality.

## Methods

### Data source

Data in this retrospective cohort study were retrieved from the electronic health records of the Hong Kong Hospital Authority (HA). The HA is a statutory administrative organization responsible for providing healthcare services to more than 7.3 million Hong Kong citizens. It manages a comprehensive network of 43 public hospitals, 49 specialty outpatient clinics, and 74 general outpatient clinics, offering more than 90% of all primary, secondary, and tertiary care services. The HA database stores a vast array of patient information, encompassing patient demographics, date of registered death, drug dispensing records, diagnoses, procedures, and laboratory test results. The data obtained from this database have been used to generate important epidemiological studies involving individuals with diabetes, particularly regarding their cardio-renal-metabolic sequelae [19–22].

### Study design and population

We included all adults with type 2 diabetes diagnosed between 1 January 2008 and 31 December 2020 who had at least one HDL-C measurement after the diagnosis of type 2 diabetes. Type 2 diabetes was identified by using the International Classification of Primary Care, Version 2 (ICPC-2) code of T90, the International Statistical Classification of Disease, 9th Revision, Clinical Modification (ICD-9-CM) codes of ‘250. × 0’ and ‘250. × 2’, or the prescription of oral anti-diabetic medications.

Individuals with history of stable coronary artery disease or intervention for stable coronary artery disease were not excluded, but those who had diagnoses of major adverse cardiovascular events (MACE; including myocardial infarction [MI], stroke, or heart failure [HF]) on or before baseline were excluded. Further exclusion criteria were (i) diagnosis of end-stage kidney disease (ESKD), defined as having estimated glomerular filtration rate (eGFR) < 15 mL/min/1.73m^2^, dialysis or kidney transplantation on or before baseline; and (ii) death within one month after the index date.

The index date was defined as the date of the first HDL-C measurement available after the diagnosis of type 2 diabetes. All included subjects were followed up from the index date to the occurrence of outcomes, death, or the end of the observation period (31 December 2020), whichever came first.

### Definition of outcomes

The primary outcome was incident MACE, a composite of MI, stroke, HF and cardiovascular mortality, adapted for use in the electronic health database in HA [23]. Secondary outcomes were (i) the incidence of individual components of MACE, (ii) all-cause mortality, and (iii) non-cardiovascular mortality. These outcome events were identified by diagnosis codes of ICPC‐2, ICD‐9‐CM, and the International Statistical Classification of Diseases and Related Health Problems, 10th Revision, Clinical Modification (ICD-10-CM) [24]. All classification codes for disease diagnosis, treatment procedures, and laboratory parameters are listed in Additional file 1: Table S1. The list of medications is summarized in Additional file 1: Table S2.

### Definition of covariates

Baseline characteristics included age, sex, duration of diabetes, smoking and drinking habits, body mass index (BMI), clinical and laboratory parameters, Charlson Comorbidity Index (CCI), pre-existing CVD (history of stable coronary artery disease, or revascularization for stable coronary artery disease), diabetic retinopathy and neuropathy, and the use of medications within the 6 months prior to baseline. Clinical and laboratory parameters included systolic blood pressure (SBP), diastolic blood pressure (DBP), glycated haemoglobin (HbA1c), fasting glucose, estimated glomerular filtration rate (eGFR), LDL-C, triglyceride and albuminuria status. Albuminuria status was categorized based on urine albumin to creatinine ratio (UACR): A1 (UACR < 3 mg/mmol), A2 (UACR ≥ 3 to ≤ 30 mg/mmol), and A3 (UACR > 30 mg/mmol).

### Data analysis

Continuous variables were presented as mean (standard deviation), and categorical variables were shown as frequency (proportion). To assess the relationship between HDL-C levels and the outcomes, eligible individuals were categorized into three groups based on their HDL-C levels: low HDL-C (≤ 40 mg/dL), medium HDL-C (> 40 and ≤ 80 mg/dL) and high HDL-C (> 80 mg/dL) groups. The Kruskal–Wallis test and Pearson’s chi-squared test were used to compare the differences in continuous variables and categorical variables across three groups, respectively.

Over the follow-up period, we calculated the cumulative incidence and crude incidence of the outcomes. The 95% confidence intervals (CIs) of the crude incidence rates were generated based on Poisson distribution. Cox proportional hazards regression models were used to identify the relationship between HDL-C levels and risks of outcomes, with the reference HDL-C category being between 40 and 80 mg/dL (medium HDL-C). Hazard ratios (HRs) with 95% CIs were calculated by using an unadjusted model (Model 1) and a model adjusted with age, sex, and index year (Model 2). To explore both linear and non-linear relationships between HDL-C as a continuous variable and incident MACE, we performed restricted cubic spline regression models. Four cut-off points, placed at the percentiles recommended by Harrell (5th, 35th, 65th, and 95th percentiles), were used with a reference point at an HDL-C level of 60 mg/dL [25].

Subgroup analyses were conducted by stratification according to the following baseline variables: age (≤ 60 vs > 60 years), sex (male vs female), statin (users vs non-users), use of any anti-diabetic medication (users vs non-users), HbA1c levels (< 8 vs ≥ 8%), eGFR (< 60 vs ≥ 60 mL/min/1.73m^2^), and presence of obesity (BMI ≤ 25 vs > 25 kg/m^2^).

Several sensitivity analyses were performed to assess the robustness of results. First, we performed complete case analysis after excluding individuals with missing baseline characteristics. Data completion rates of baseline characteristics are shown in Additional file 1: Table S3. A Cox proportional hazards regression model adjusting for all baseline covariates and restricted cubic spline regression models were performed to investigate the associations between HDL-C levels and adverse outcomes. Second, we restricted the analyses to individuals who had > 1 year of follow-up. Third, we repeated the analysis in individuals without pre-existing CVD. Fourth, we applied sex-specific cut-off for low HDL-C levels, where male with HDL-C < 40 mg/dL and female with HDL-C < 50 mg/dL were considered to have ‘low HDL-C’. Male with levels ranging from 40 to 80 mg/dL and female with levels from 50 to 80 mg/dL were classified as having 'medium HDL-C'. Those with levels exceeding 80 mg/dL were categorized as having 'high HDL-C'. We conducted Cox proportional hazards regression models separately for male and female [26]. Last but not least, we evaluated the association between HDL-C levels and incident three-point MACE (composite of MI, stroke and cardiovascular mortality), i.e. omitting HF in the composite outcome [27].

A two-tailed *P* < 0.05 was considered statistically significant. Statistical analysis was performed by using Stata Version 16.0 (StataCorp LP, College Station, TX) and R Version 4.3.0. The analyses were conducted by LLL and analyzed independently by XDL for quality assurance.

## Results

**Fig. 1:**
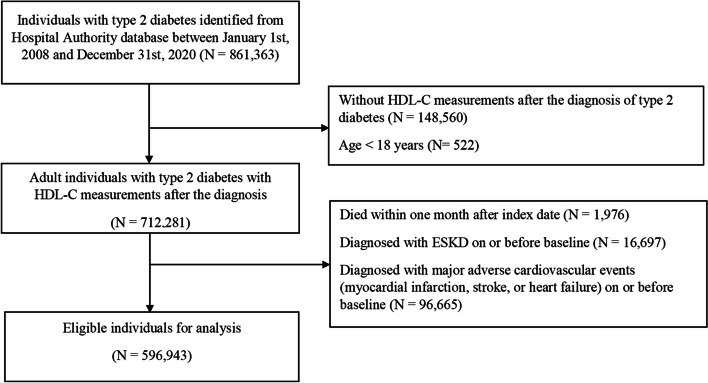
Flowchart of Individuals with Type 2 Diabetes Included and Excluded for this Analysis. HDL-C = High-density lipoprotein cholesterol; ESKD = End-stage kidney disease. The index date was defined as the date of the first HDL-C measurement available after the diagnosis of type 2 diabetes

A total of 596,943 individuals with type 2 diabetes were identified (Fig. 1). Among them, 168,931 (28.30%), 412,863 (69.16%), and 15,149 (2.54%) individuals were assigned to low HDL-C, medium HDL-C, and high HDL-C groups, respectively. The mean age was 62.0 years. There was no sex preponderance. The mean BMI was 26.0 kg/m^2^ and HbA1c was 7.6%. Of note, 29.58% were statin users. The comparison of the three HDL-C categories is shown in Table 1. Individuals in the high HDL-C group were older and more likely female, non-smoker and non-drinker. Regarding clinical and laboratory parameters, individuals in the high HDL-C group had lower levels of triglyceride, HbA1c and fasting glucose. Less individuals in the high HDL-C group were statin users (25.20%). More individuals in the low HDL-C group had albuminuria categories A2/3.

**Table 1:** Baseline characteristics of individuals in low, medium, and high HDL-C groups

	HDL-C levels^a^				
	Overall (*N* = 596,943)	Low HDL-C (*N* = 168,931)	Medium HDL-C (*N* = 412,863)	High HDL-C (*N* = 15,149)	*P*-value
	Mean ± SD/N (%)	Mean ± SD/N (%)	Mean ± SD/N (%)	Mean ± SD/N (%)	
**Socio-demographics**					
Sex					< 0.001
Male	299,398 (50.16)	112,339 (66.50)	182,375 (44.17)	4,684 (30.92)	
Female	297,545 (49.84)	56,592 (33.50)	230,488 (55.83)	10,465 (69.08)	
Age, year	62.0 ± 12.3	60.5 ± 13.0	62.6 ± 12.0	65.1 ± 12.3	< 0.001
Smoking status					< 0.001
Non-smoker	395,758 (66.30)	93,417 (55.30)	290,829 (70.44)	11,512 (75.99)	
Ex-smoker	85,888 (14.39)	29,307 (17.35)	54,971 (13.31)	1,610 (10.63)	
Current smoker	73,750 (12.35)	32,168 (19.04)	40,588 (9.83)	994 (6.56)	
Alcohol status					< 0.001
Non-drinker	351,987 (58.96)	92,145 (54.55)	250,571 (60.69)	9,271 (61.20)	
Ex-drinker	36,407 (6.10)	13,022 (7.71)	22,706 (5.50)	679 (4.48)	
Current drinker	99,028 (16.59)	30,594 (18.11)	66,333 (16.07)	2,101 (13.87)	
**Clinical and laboratory parameters**					
Body mass index, kg/m^2^	26.0 ± 4.3	26.8 ± 4.3	25.8 ± 4.2	23.1 ± 4.2	< 0.001
HbA1c					< 0.001
%	7.6 ± 1.9	7.9 ± 2.0	7.5 ± 1.8	7.3 ± 1.9	
mmol/mol	60.0 ± 20.8	63.0 ± 21.9	58.0 ± 19.7	56.0 ± 20.8	
Fasting glucose, mmol/L	7.9 ± 2.8	8.3 ± 3.0	7.8 ± 2.7	7.6 ± 2.9	< 0.001
Systolic blood pressure, mmHg	135.6 ± 18.1	136.1 ± 18.4	135.5 ± 18.0	135.1 ± 18.9	< 0.001
Diastolic blood pressure, mmHg	76.5 ± 11.2	77.9 ± 11.5	76.1 ± 11.0	73.6 ± 10.9	< 0.001
LDL-C, mmol/L	2.9 ± 0.9	2.8 ± 0.9	3.0 ± 0.9	2.7 ± 1.0	< 0.001
Triglyceride, mmol/L	1.7 ± 1.3	2.3 ± 1.8	1.5 ± 0.9	0.9 ± 0.5	< 0.001
eGFR, mL/min/1.73m^2^	83.3 ± 20.9	82.2 ± 21.9	83.7 ± 20.5	83.0 ± 19.1	< 0.001
Albuminuria status					< 0.001
UACR < 3 mg/mmol	317,960 (53.26)	82,029 (48.56)	227,798 (55.18)	8,133 (53.69)	
UACR 3–30 mg/mmol	110,483 (18.51)	34,180 (20.23)	73,936 (17.91)	2,367 (15.62)	
UACR > 30 mg/mmol	27,547 (4.61)	9,725 (5.76)	17,161 (4.16)	661 (4.36)	
Duration of diabetes, year	3.0 ± 4.6	3.1 ± 4.6	3.0 ± 4.6	3.5 ± 5.2	< 0.001
CCI	4.2 ± 1.7	4.1 ± 1.8	4.2 ± 1.6	4.5 ± 1.7	< 0.001
Pre-existing cardiovascular diseases	38,100 (6.4)	13,485 (8.0)	24,051 (5.8)	564 (3.7)	< 0.001
Diabetic complications					
Retinopathy	8,321 (1.39)	2,437 (1.44)	5,670 (1.37)	214 (1.41)	0.121
Neuropathy	3,280 (0.55)	1,137 (0.67)	2,044 (0.50)	99 (0.65)	< 0.001
**Use of medication (6 months prior to baseline)**					
Anti-diabetic medications	456,582 (76.49)	138,481 (81.97)	307,766 (74.54)	10,335 (68.22)	< 0.001
Oral anti-diabetic medications	448,684 (75.16)	135,909 (80.45)	302,953 (73.38)	9,822 (64.84)	< 0.001
Metformin	398,542 (66.76)	119,685 (70.85)	270,456 (65.51)	8,401 (55.46)	< 0.001
Sulfonylurea	220,823 (36.99)	73,826 (43.70)	142,458 (34.50)	4,539 (29.96)	< 0.001
Thiazolidinedione	3,692 (0.62)	1,209 (0.72)	2,385 (0.58)	98 (0.65)	< 0.001
SGLT2i	1,211 (0.20)	422 (0.25)	764 (0.19)	25 (0.17)	< 0.001
GLP1rA	144 (0.02)	50 (0.03)	93 (0.02)	1 (0.01)	0.107
DPP4i	6,222 (1.04)	2,277 (1.35)	3,799 (0.92)	146 (0.96)	< 0.001
Alpha-glucosidase inhibitors	2,723 (0.46)	1,044 (0.62)	1,624 (0.39)	55 (0.36)	< 0.001
Insulin	46,141 (7.73)	18,327 (10.85)	26,314 (6.37)	1,500 (9.90)	< 0.001
Anti-hypertensive medications	404,510 (67.76)	117,172 (69.36)	278,000 (67.33)	9,338 (61.64)	< 0.001
ACEI/ARB	204,121 (34.19)	63,914 (37.83)	135,960 (32.93)	4,247 (28.03)	< 0.001
Beta-blockers	151,852 (25.44)	51,161 (30.29)	98,335 (23.82)	2,356 (15.55)	< 0.001
Calcium-channel blockers	260,926 (43.71)	73,047 (43.24)	181,609 (43.99)	6,270 (41.39)	< 0.001
Diuretics	63,545 (10.65)	19,364 (11.46)	42,571 (10.31)	1,610 (10.63)	< 0.001
Other anti-hypertensive medications	40,960 (6.86)	13,745 (8.14)	26,386 (6.39)	829 (5.47)	< 0.001
Anticoagulants	7,950 (1.33)	3,293 (1.95)	4,517 (1.09)	140 (0.92)	< 0.001
Antiplatelets	72,053 (12.07)	25,266 (14.96)	45,489 (11.02)	1,298 (8.57)	< 0.001
Lipid-lowering agents	192,156 (32.19)	52,308 (30.96)	135,879 (32.91)	3,969 (26.20)	< 0.001
Statins	176,591 (29.58)	43,963 (26.02)	128,810 (31.20)	3,818 (25.20)	< 0.001
Fibrates	18,684 (3.13)	9,879 (5.85)	8,638 (2.09)	167 (1.10)	< 0.001
Ezetimibe	1,110 (0.19)	335 (0.20)	756 (0.18)	19 (0.13)	0.102
Other lipid-lowering agents	338 (0.06)	121 (0.07)	201 (0.05)	16 (0.11)	< 0.001
NSAIDs	71,565 (11.99)	20,564 (12.17)	49,380 (11.96)	1,621 (10.70)	< 0.001

*SD* standard deviation, *HbA1c* Hemoglobin A1c, *LDL-C* Low-density lipoprotein cholesterol, *HDL-C* High-density lipoprotein cholesterol, *eGFR* Estimated glomerular filtration rate, *UACR* Urine Albumin-Creatinine Ratio, *CCI* Charlson Comorbidity Index, *DPP4i* Dipeptidyl peptidase 4 inhibitors, *SGLT2i* Sodium-glucose cotransporter 2 inhibitors, *GLP1rA* Glucagon like peptide 1 receptor agonists, *ACEI/ARB* Angiotensin-converting enzyme inhibitors / Angiotensin receptor blockers, *NSAIDs* Non-steroidal anti-inflammatory drugs

^a^HDL-C groups were classified by HDL-C levels: low HDL-C (≤ 40 mg/dL), medium HDL-C (> 40 and ≤ 80 mg/dL) and high HDL-C (> 80 mg/dL)

### Association between HDL-C levels (Stratified by Categories) and Incident MACE

Upon a median follow-up of 79.5 months (interquartile range: 35.5 – 117.5 months), there were 81,592 MACEs (incidence rate of 2.08 events per 100 person-years), specifically 21,838 events of MI, 47,201 events of stroke, 31,073 events of HF, and 1,788 events of cardiovascular mortality. Table 2 presents the number of events and crude incidence rate of MACE in the three HDL-C groups.

**Table 2:** Hazard ratios and crude incidence rate of MACEs for low HDL-C and high HDL-C groups compared with medium HDL-C group

					Model1^e^			Model2^f^		
HDL-C levels^a^	Event	Crude incidence rate^b^	95% CI^c^	Person-years	HR^d^	95% CI	*P*-value	HR^d^	95% CI	*P*-value
Low HDL-C	26,925	2.42	(2.39, 2.45)	1,111,501	1.244	(1.225, 1.262)	< 0.001	1.254	(1.235, 1.273)	< 0.001
Medium HDL-C	52,630	1.94	(1.93, 1.96)	2,709,163	Ref			Ref		
High HDL-C	2037	2.10	(2.01, 2.20)	96,912	1.085	(1.038, 1.134)	< 0.001	1.064	(1.018, 1.112)	0.006

*MACEs* Major adverse cardiovascular events, *HDL-C* High-density lipoprotein cholesterol, *HR* Hazard ratio, *CI* Confidence interval

^a^HDL-C groups were classified by HDL-C levels: low HDL-C (≤ 40 mg/dL), medium HDL-C (> 40 and ≤ 80 mg/dL) and high HDL-C (> 80 mg/dL)

^b^The unit of crude incidence rate: events per 100 person-years

^c^The 95% CIs of the crude incidence rates were generated according to the Poisson distribution

^d^HR > 1 (or < 1) indicates low/high HDL-C group had higher (lower) risk of MACE outcomes compared to medium HDL-C groups

^e^Model1: model without adjustment

^f^Model2: model adjusted for age, sex, and index year

In the unadjusted analysis, both low HDL-C group (HR 1.244, 95% CI 1.225–1.262; *P* < 0.001) and high HDL-C group (HR 1.085, 95% CI 1.038–1.134; *P* < 0.001) were associated with a higher risk of developing MACE compared to the medium HDL-C group. In the analysis adjusted for age, sex, and index year, consistent findings were obtained for low and high HDL-C group when compared to medium HDL-C group (HR, 1.254; 95% CI, 1.235–1.273; *P* < 0.001; HR, 1.064; 95% CI, 1.018–1.112; *P* = 0.006, respectively). Of note, the hazard ratio associated with low HDL-C group was higher than that associated with high HDL-C group.

### Association between HDL-C levels (as Continuous Variable) and incident MACE

**Fig. 2:**
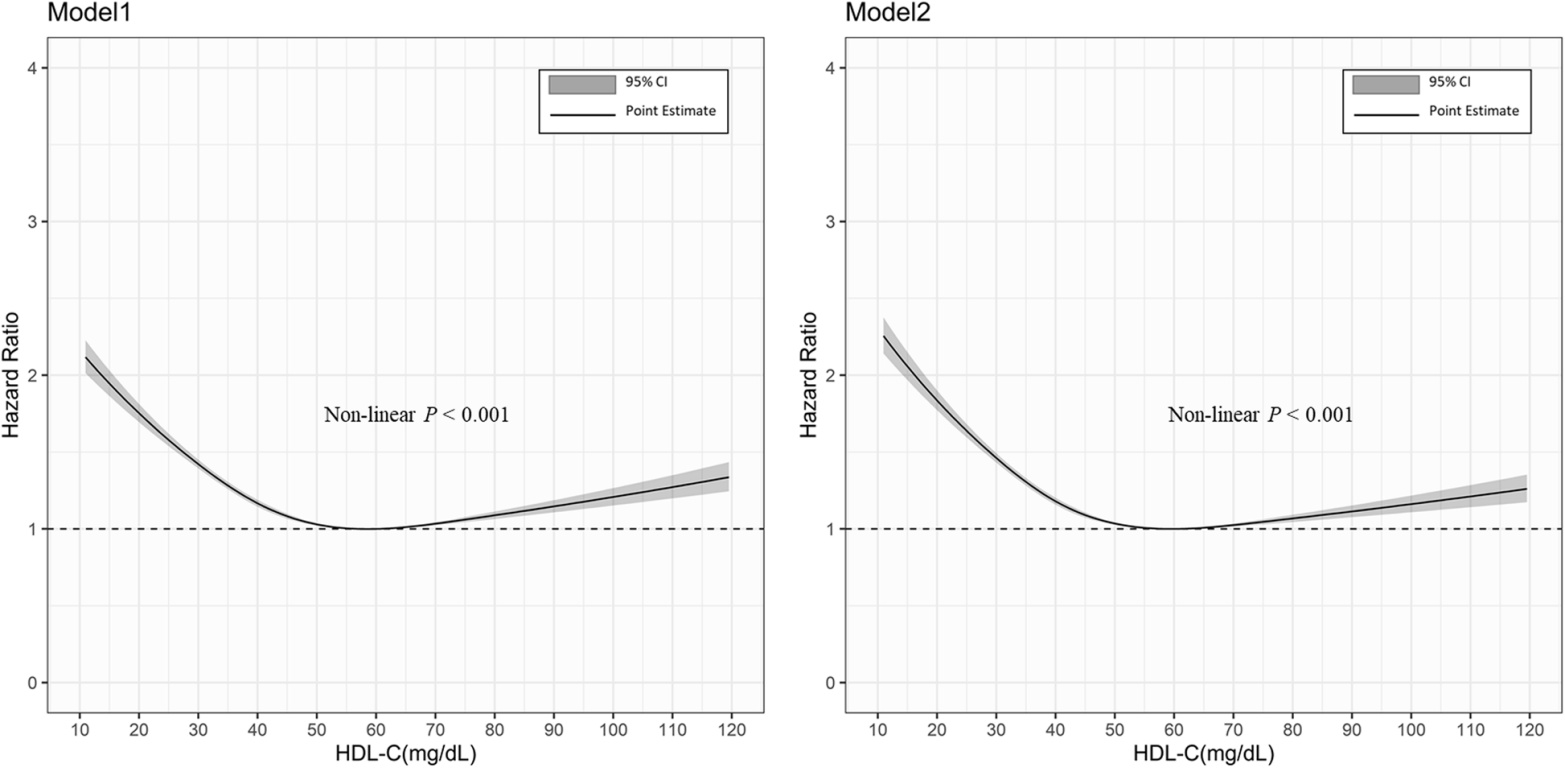
Association between Continuous HDL-C levels and Incident MACEs Estimated by Restricted Cubic Splines Models. **a** without adjustment (Model 1), and (**b**) with adjustment for age, sex, and index year (Model 2); MACEs = Major adverse cardiovascular events; HDL-C = High-density lipoprotein cholesterol; CI = Confidence interval; The reference point was set at the HDL-C level of 60 mg/dL

The spline curves of the Cox regression models with or without adjustment for covariates both showed a U-shaped relationship between the HDL-C level and the risk of incident MACE (Fig. 2). Consistent with the analyses using categorized HDL-C levels, the hazard ratio associated with the lower spectrum of HDL-C levels was higher than that associated with the higher spectrum of HDL-C levels.

### Analyses of secondary outcomes

**Fig. 3:**
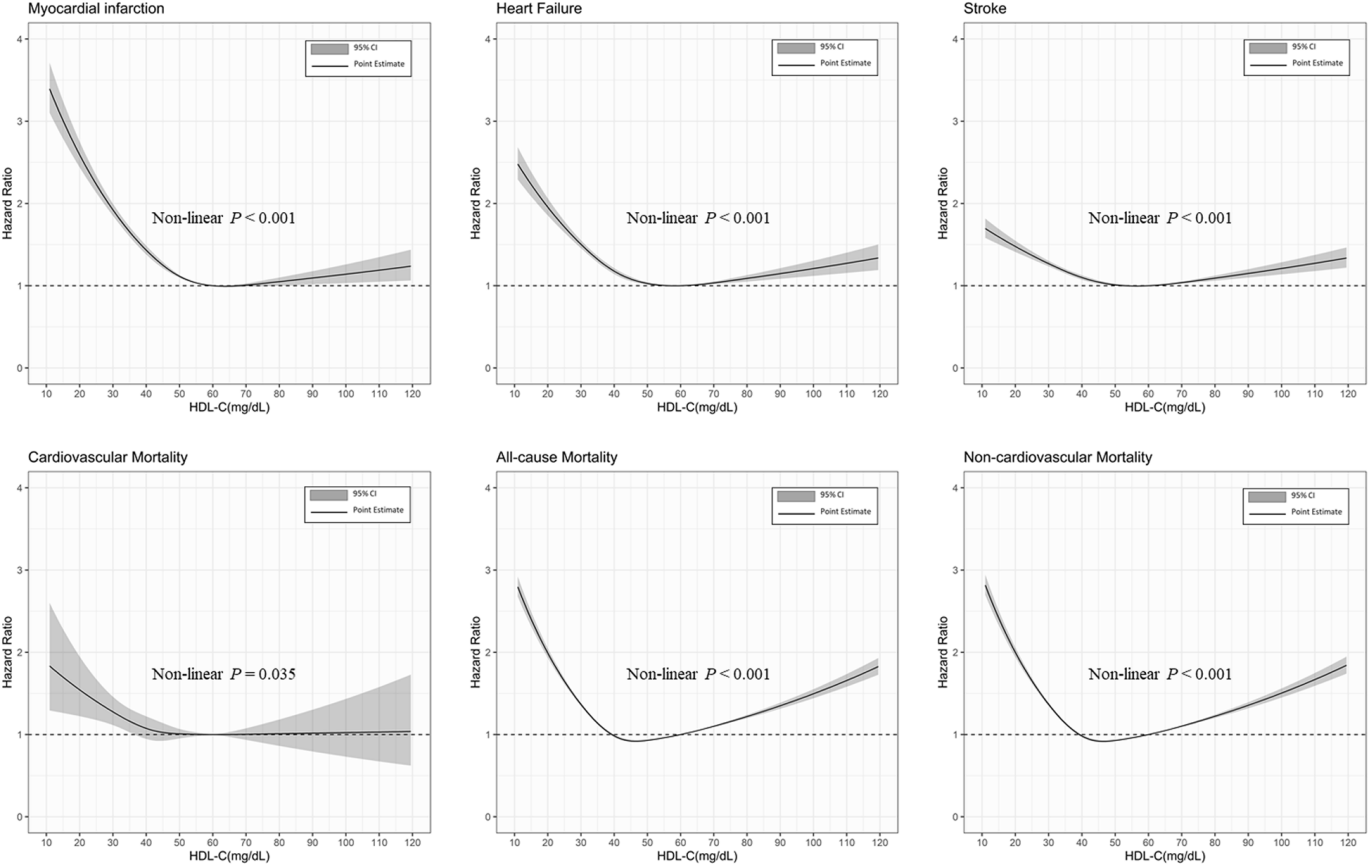
Association between Continuous HDL-C Levels and Incident Secondary Outcomes Estimated by Restricted Cubic Spline Models. HDL-C = High-density lipoprotein cholesterol; CI = Confidence interval. The reference point was set at the HDL-C level of 60 mg/dL

Both low and high HDL-C groups were associated with significantly increased risks of stroke, HF, non-cardiovascular mortality and all-cause mortality compared to the medium HDL-C group (all *p* < 0.05). However, the significantly increased risks of myocardial infarction and cardiovascular mortality were observed only in the low HDL-C group relative to the medium HDL-C group, with a hazard ratio (HR) of 1.441 (95% CI, 1.390–1.494; *P* < 0.001) for myocardial infarction and an HR of 1.193 (95% CI, 1.032–1.380; *P* = 0.017) for cardiovascular mortality (Additional file 1: Table S4). No significant associations were found for the high HDL-C group. Restricted spline curves also showed the similar findings (Fig. 3).

### Subgroup analyses

Spline plots in Additional file 1: Fig. S1 indicate a U-shaped association between HDL-C levels and MACE in most subgroups (stratified by sex, statin use, anti-diabetic medication use, eGFR, HbA1c levels, and presence of obesity). A non-linear relationship between high HDL-C levels and MACE can be seen across different age subgroups (all non-linear *p* < 0.001). Notably, association between high HDL-C levels and MACE were not statistically significant in those ≤ 60 years old. Moreover, both males and females exhibit a U-shaped relationship between HDL-C levels and MACE (all non-linear *p* < 0.001), with this trend being more pronounced in males.

### Sensitivity analyses

In the complete case analysis (Additional file 1: Table S5), when compared to medium HDL-C group, both the low and high HDL-C groups exhibited higher risks of developing MACE (HR, 1.075; 95% CI, 1.048–1.103; *P* < 0.001; HR, 1.162; 95% CI, 1.087–1.242; *P* < 0.001, respectively) in the fully adjusted models considering sociodemographic (age, sex, smoking and alcohol status), clinical and laboratory parameters, comorbidities and baseline medications (Additional file 1: Table S6). The U-shaped curves between HDL-C levels and MACE and secondary outcomes were consistently seen as in the main analysis after applying restricted cubic spline regression models and fully adjusting for all baseline covariates listed in Table 1 in individuals with complete baseline covariates (Additional file 1: Fig. S2 and Fig. S3).

Upon restriction of analysis to the 545,819 individuals with type 2 diabetes who had follow-up > 1 year, the U-shaped association between HDL-C levels and incident MACE remained consistent (Additional file 1: Fig. S4).

Similarly, upon restriction of analysis to the 558,843 individuals without pre-existing CVD, the U-shaped curve was still seen (Additional file 1: Fig. S5).

The results of Cox proportional analyses for sex-specific categories of HDL-C levels are shown in Additional file 1: Table S7 and Table S8. Consistent with our main findings, both low and high HDL-C levels are associated with a higher risk of developing MACE compared to the medium HDL-C group among different sex groups. Specifically, in males, the HRs are 1.237 (95% CI, 1.212–1.261; *P* < 0.001) for low HDL-C and 1.128 (95% CI, 1.045–1.217; *P* = 0.002) for high HDL-C. In females, the corresponding HRs are 1.183 (95% CI, 1.160–1.208; *P* < 0.001) for low HDL-C and 1.079 (95% CI, 1.021–1.141; *P* = 0.007) for high HDL-C.

Similar results were observed when analyzing 3-point MACEs as outcomes. The HRs for the low and high HDL-C groups compared to the medium HDL-C group were 1.242 (*P* < 0.001) and 1.063 (*P* = 0.016), respectively (Additional file 1: Table S9). The U-shaped curves between HDL-C levels and 3-point MACE were consistently seen both before and after adjustment (Additional file 1: Fig. S6).

## Discussion

Our population-based study of close to 0.6 million individuals with type 2 diabetes demonstrated a U-shaped relationship between HDL-C levels and incident MACE. This U-shaped relationship was largely consistent across subgroups. The exceptions were that the association did not reach statistical significance in the younger age group, and the exaggeration of the U-shaped relationship among male compared with female. Our results suggested the need to review the role of HDL-C in the cardiovascular risk prediction in type 2 diabetes. High HDL-C did not protect one from MACE and was even associated with worse cardiovascular outcomes. Even the widespread use of statins did not modify the U-shaped relationship. In fact, the U-shaped relationship also applied to non-cardiovascular and all-cause mortality. Further evaluations of the underlying mechanisms of the worse outcomes among individuals with type 2 diabetes and high HDL-C levels are warranted.

Recent studies in the general populations suggest that the association between HDL-C levels and cardiovascular events may not be linear across the entire range but follows a U-shaped relationship [10, 28]. In line with these, previous clinical trials of therapies which substantially elevate HDL-C levels did not result in lower risks of cardiovascular events [6, 29, 30]. In contrast to the general population, less is studied regarding the implication of high HDL-C levels and various outcomes specifically in type 2 diabetes. Our findings based on a large cohort of close to 0.6 million individuals with type 2 diabetes showed a U-shaped relationship between HDL-C levels and adverse outcomes, particularly cardiovascular outcomes. Several cohort studies have studied the correlation between very high HDL-C levels and adverse outcomes in type 2 diabetes, as part of these studies [16, 17]. Ishibashi et al. evaluated a population derived from JMDC Claims Database and demonstrated a U-shaped relationship between HDL-C levels and cardiovascular events (MI, stroke and all-cause mortality) in a subset of 56,881 individuals with diabetes defined by fasting glucose. Of note, the JMDC Claims Database comprised mainly working-age Japanese population. Furthermore, individuals with prior CVD, use of lipid-lowering and anti-diabetic medications were excluded from the analysis, limiting the generalizability of results [17]. In another study by Wu et al., among the 8244 Chinese individuals with diabetes included, while high HDL-C levels (> 80 mg/dL) were associated with higher risk of CVD, low HDL-C levels did not predict future cardiovascular risks [16]. The study, however, was limited by the lack of HbA1c measurement to define diabetes and the male predominance. Our study extended the current understanding by evaluating a diverse cohort of individuals with type 2 diabetes, showing a U-shaped relationship between HDL-C levels and MACE stratified by sex, statin use, use of anti-diabetic medications, eGFR and HbA1c levels and the presence of obesity. In the sex-specific analyses, the increase in hazard ratio associated with high HDL-C levels was higher in males than in females. This was similarly observed in a previous study using the UK Biobank cohort [12]. The precise mechanism is unclear, although the intrinsically higher cardiovascular risk in men may provide partial explanation. A few secondary analyses did not reach statistical significance: (i) the association between high HDL-C levels and incident MACE in the younger age group, and (ii) the associations of high HDL-C levels with MI and cardiovascular mortality. That could be contributed by the relatively lower number of events in these secondary analyses.

The association between high HDL-C levels and adverse cardiovascular outcomes is generalizable to the large population of individuals with type 2 diabetes, which may bear implications in the construction of cardiovascular risk calculators as current risk calculators (including the SCORE2-Diabetes [31]) consider HDL-C level as a linear variable and may not account for the higher cardiovascular risks linked to the high HDL-C levels. It is also interesting to note that the excess risk of MACE associated with high HDL-C levels was less than that associated with low HDL-C levels. This asymmetry of the U-shaped association between HDL-C levels and MACE has been suggested in some [11, 17], but not all [12], studies. Further population-based epidemiological studies would help to confirm such observation to refine cardiovascular risk estimation.

There are some postulated mechanisms for the elevated cardiovascular risks at high HDL-C levels in type 2 diabetes. Very high HDL-C levels may be a result of genetic variants that have detrimental effects on disease susceptibility. For instance, certain genetic mutations in LIPC and SCARB1, which have been linked to a higher risk of coronary heart disease [32, 33]. Moreover, HDL-C levels represent the overall concentration of HDL-C in the circulation, which is a mix of HDL particles with different biological functions. HDL particles possess multiple biological activities which can influence glycaemia, immunity and inflammation, atherosclerosis and function of vascular cells [34]. HDL consists of a family of lipoprotein particles which differ in density, size, charge, protein and lipid composition, and HDL particles are highly heterogeneous [35]. It has been shown that various cellular functions of HDL only weakly correlated with each other. Different cellular functions of HDL were determined by different structural components [36]. Hence, HDL-C levels only reflect the cholesterol contents of the HDL pool and do not predict its composition or function. Type 2 diabetes is associated with various modifications in the structures and functions of HDL particles. For example, increased glycation/glycoxidation of HDL may impair its reverse cholesterol transport ability and diminish its antiatherogenic capacity [37]. The oxidative environment of the acute phase response in diabetes is associated with change of HDL particles from anti-inflammatory to pro-inflammatory [36, 38, 39]. The increased particle sizes and detrimental subspecies such as HDL containing apoC3 in individuals with high levels of HDL-C have been found to result in cholesterol deposition and atherosclerosis development, thereby increasing the risk of cardiovascular events [40, 41].

Interestingly, we showed that high HDL-C levels were also associated with higher all-cause and non-cardiovascular mortality in type 2 diabetes. Previous studies have reported a higher risk of mortality among individuals with high HDL-C levels in the general population [42], notably two large cohorts in the United States [43] and Canada [10], though the relationship between HDL-C levels and mortality was not evaluated specifically among the smaller subgroup with diabetes. Taken together, all these results suggest the need for further mechanistic work to elucidate the reason for the adverse cardiovascular and non-cardiovascular outcomes associated with high HDL-C levels in individuals with type 2 diabetes. For example, whether HDL particle size plays a role in such associations in type 2 diabetes deserves further evaluations. A recent study using the UK Biobank revealed that the increased risk of mortality associated with very high HDL-C levels existed only in individuals with hypertension, but not in those without hypertension, and that the increased risk at high HDL-C levels in hypertension was likely driven by larger HDL particles [44].

We observed a lower proportion of current alcohol users in the high HDL-C group at baseline, which appeared to contrast with the general observation that alcohol consumption increases HDL-C levels. Nonetheless, in the multivariable linear regression analysis adjusted for age, sex and BMI, current alcohol users indeed had higher levels of baseline HDL-C levels (β = 1.48, *p* < 0.001), yielding a consistent finding with the general observation.

The strength of our study lies in the large sample size of close to 0.6 million individuals with type 2 diabetes who are well characterized, and the relatively long follow-up of the cohort. Nonetheless, our results should be interpreted acknowledging certain limitations. First, despite our efforts to account for a wide range of confounding factors and in performing sensitivity analyses, some residual confounders (e.g. lifestyle factors) that could influence the studied associations were not captured in the electronic health records. Second, the study population was predominantly Chinese, limiting the generalizability of our findings to other ethnicities. Third, our study included exclusively individuals with type 2 diabetes, and therefore, whether our findings apply to individuals with type 1 diabetes remains to be evaluated. Fourth, we did not have a control group of individuals without diabetes. Last but not least, an observational study could not establish a causal association between high HDL-C levels and increased cardiovascular risk.

## Conclusions

In conclusion, our study demonstrated a U-shaped association between HDL-C levels and incident MACE in a broad spectrum of individuals with type 2 diabetes. The results highlight the need to attend to the cardiovascular risk profile of individuals with type 2 diabetes who have high HDL-C levels, and for mechanistic studies for the increased cardiovascular risks observed at high HDL-C levels in type 2 diabetes.

## Supplementary Information

Additional file 1: Fig. S1. Association between continuous HDL-C levels and incident MACEs estimated by restricted cubic splines models among subgroups. Fig. S2. Restricted cubic splines models for incident MACEs with adjustment for all baseline covariates. Fig. S3. Restricted cubic splines models for secondary outcomes with adjustment for all baseline covariates. Fig. S4. Restricted cubic splines models for incident MACEs among individuals with more than 1-year follow-up. Fig. S5. Restricted cubic splines models for incident MACEs among individuals without any pre-existing cardiovascular diseases and procedures. Fig. S6. Restricted cubic splines models for incident 3-point MACEs among individuals without any pre-existing cardiovascular diseases and procedures. Table S1. List of disease diagnosis codes, procedure codes, and laboratory criteria for each clinical diagnosis. Table S2. List of drug items and identify codes. Table S3. Data completion rate. Table S4. Hazard ratios and crude incidence rate of secondary outcomes for low HDL-C and high HDL-C groups compared with medium HDL-C group. Table S5. Baseline characteristics for individuals without missing covariates and stratified by different HDL-C categories. Table S6. Hazard ratios and crude incidence rate of MACEs among individuals without missing covariates. Table S7. Hazard ratios and crude incidence rate of MACEs for low HDL-C and high HDL-C groups compared with medium HDL-C group for male. Table S8. Hazard ratios and crude incidence rate of MACEs for low HDL-C and high HDL-C groups compared with medium HDL-C group for female. Table S9. Hazard ratios and crude incidence rate of 3-point MACEs for low HDL-C and high HDL-C groups compared with medium HDL-C group.

## Data Availability

The data of this study were provided by the Hong Kong Hospital Authority. Restrictions apply to the availability of these data, which were used under license.

## Abbreviations

HDL-C: High-density lipoprotein cholesterol
CVD: Cardiovascular diseases
MACE: Major adverse cardiovascular events
CETP: Cholesteryl ester transfer protein
LDL-C: Low-density lipoprotein cholesterol
HA: Hospital Authority
ICPC-2: International Classification of Primary Care, Version 2
ICD-9-CM: International Statistical Classification of Disease, 9th Revision, Clinical Modification
MI: Myocardial infarction
HF: Heart failure
ESKD: End-stage kidney disease
eGFR: Estimated glomerular filtration rate
ICD-10-CM: International Statistical Classification of Diseases and Related Health Problems, 10th Revision, Clinical Modification
BMI: Body mass index
CCI: Charlson Comorbidity Index
SBP: Systolic blood pressure
DBP: Diastolic blood pressure
HbA1c: Hemoglobin A1c
UACR: Urine albumin to creatinine ratio
CIs: Confidence intervals
HR: Hazard ratio
