# Supplementary material for: The association of HDL-cholesterol levels with incident major adverse cardiovascular events and mortality in 0.6 million individuals with type 2 diabetes: a population-based retrospective cohort study
Source: BMC Med. 2024 Dec 18;22:586. doi: 10.1186/s12916-024-03810-4 (PMC11657474; doi:10.1186/s12916-024-03810-4)
Supplement: Supplementary file 1 — Additional file 1: Fig. S1. Association between continuous HDL-C levels and incident MACEs estimated by restricted cubic splines models among subgroups. Fig. S2. Restricted cubic splines models for incident MACEs with adjustment for all baseline covariates. Fig. S3. Restricted cubic splines models for secondary outcomes with adjustment for all baseline covariates. Fig. S4. Restricted cubic splines models for incident MACEs among individuals with more than 1-year follow-up. Fig. S5. Restricted cubic splines models for incident MACEs among individuals without any pre-existing cardiovascular diseases and procedures. Fig. S6. Restricted cubic splines models for incident 3-point MACEs among individuals without any pre-existing cardiovascular diseases and procedures. Table S1. List of disease diagnosis codes, procedure codes, and laboratory criteria for each clinical diagnosis. Table S2. List of drug items and identify codes. Table S3. Data completion rate. Table S4. Hazard ratios and crude incidence rate of secondary outcomes for low HDL-C and high HDL-C groups compared with medium HDL-C group. Table S5. Baseline characteristics for individuals without missing covariates and stratified by different HDL-C categories. Table S6. Hazard ratios and crude incidence rate of MACEs among individuals without missing covariates. Table S7. Hazard ratios and crude incidence rate of MACEs for low HDL-C and high HDL-C groups compared with medium HDL-C group for male. Table S8. Hazard ratios and crude incidence rate of MACEs for low HDL-C and high HDL-C groups compared with medium HDL-C group for female. Table S9. Hazard ratios and crude incidence rate of 3-point MACEs for low HDL-C and high HDL-C groups compared with medium HDL-C group. [file 12916_2024_3810_MOESM1_ESM.docx]

**Additional file 1**

**Fig. S1.** Association between continuous HDL-C levels and incident MACEs estimated by restricted cubic splines models among subgroups.

**Fig. S2.** Restricted cubic splines models for incident MACEs with adjustment for all baseline covariates.

**Fig. S3.** Restricted cubic splines models for secondary outcomes with adjustment for all baseline covariates.

**Fig. S4.** Restricted cubic splines models for incident MACEs among individuals with more than 1-year follow-up.

**Fig. S5.** Restricted cubic splines models for incident MACEs among individuals without any pre-existing cardiovascular diseases and procedures.

**Fig. S6.** Restricted cubic splines models for incident 3-point MACEs among individuals without any pre-existing cardiovascular diseases and procedures.

**Table S1.** List of disease diagnosis codes, procedure codes, and laboratory criteria for each clinical diagnosis.

**Table S2.** List of drug items and identify codes.

**Table S3.** Data completion rate.

**Table S4.** Hazard ratios and crude incidence rate of secondary outcomes for low HDL-C and high HDL-C groups compared with medium HDL-C group.

**Table S5.** Baseline characteristics for individuals without missing covariates and stratified by different HDL-C categories.

**Table S6.** Hazard ratios and crude incidence rate of MACEs among individuals without missing covariates.

**Table S7.** Hazard ratios and crude incidence rate of MACEs for low HDL-C and high HDL-C groups compared with medium HDL-C group for male.

**Table S8.** Hazard ratios and crude incidence rate of MACEs for low HDL-C and high HDL-C groups compared with medium HDL-C group for female.

**Table S9.** Hazard ratios and crude incidence rate of 3-point MACEs for low HDL-C and high HDL-C groups compared with medium HDL-C group.

Fig. S1. Association between continuous HDL-C levels and incident MACEs estimated by restricted cubic splines models among subgroups.


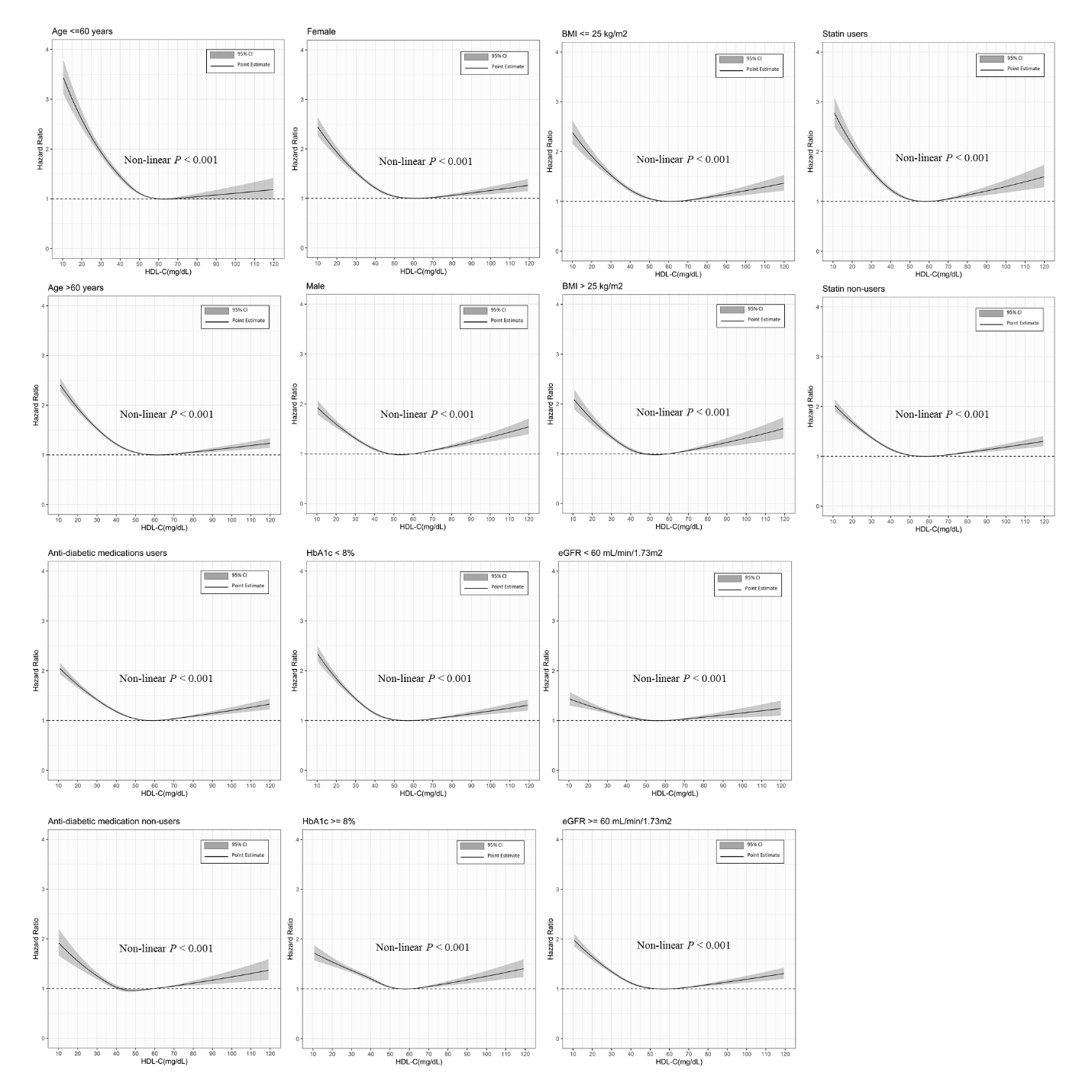


MACEs = Major adverse cardiovascular events; HDL-C = High-density lipoprotein cholesterol; HbA1c = Glycated hemoglobin; CI = Confidence interval. The reference point was set at the HDL-C level of 60 mg/dL.

Fig. S2. Restricted cubic splines models for incident MACEs with adjustment for all baseline covariates.


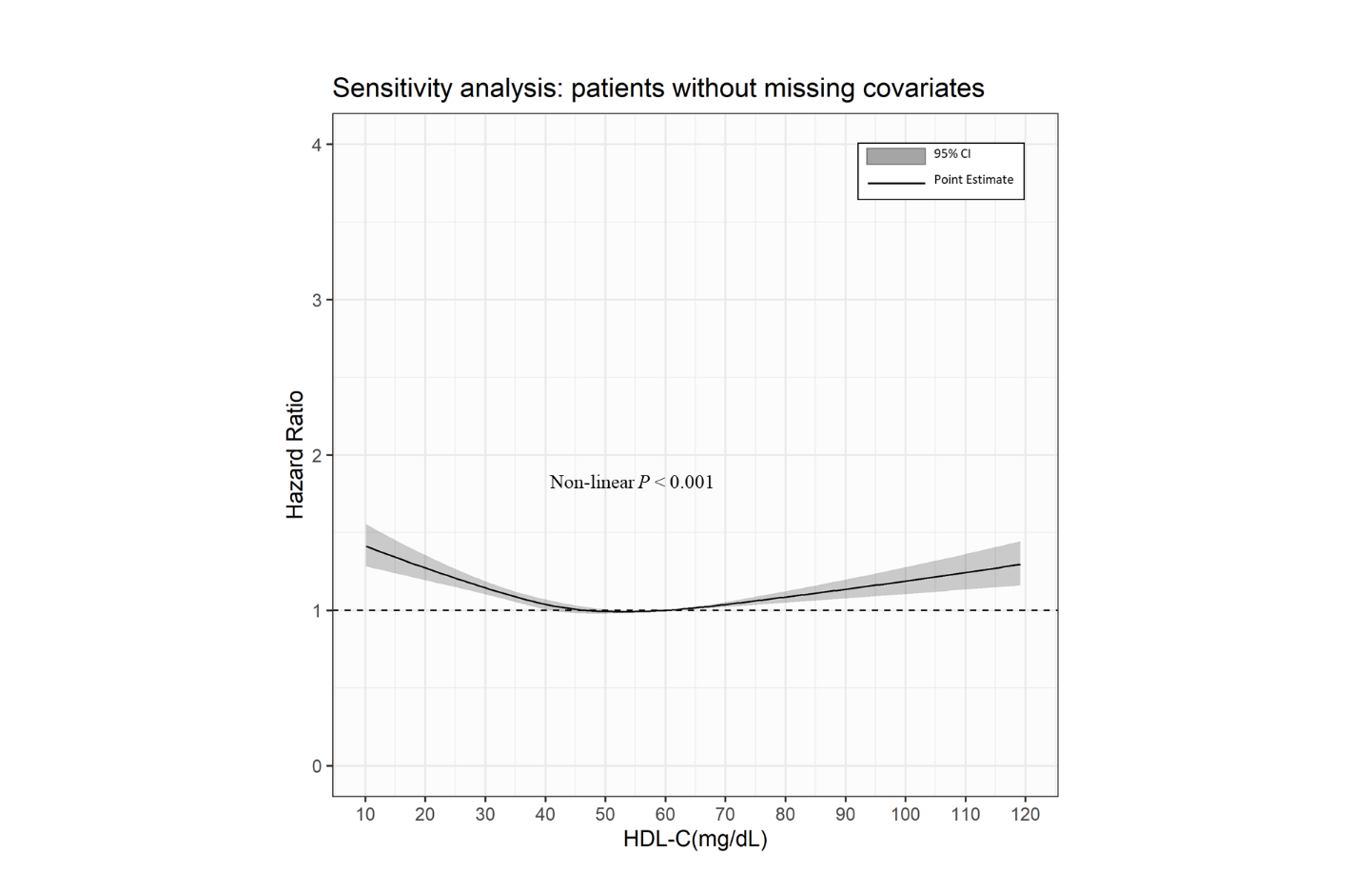


MACEs = Major adverse cardiovascular events; HDL-C = High-density lipoprotein cholesterol; CI = Confidence interval; Association between continuous HDL-C levels and incident MACE estimated by restricted cubic splines models with adjustment for all baseline covariates. The reference point was set at the HDL-C level of 60 mg/dL; Hazard ratio is calculated after adjusting for covariates, including sociodemographic characteristics (age, sex, smoking status, and alcohol status), clinical and laboratory parameters (BMI, HbA1c, fasting glucose, systolic blood pressure, diastolic blood pressure, LDL-C, triglyceride, eGFR, albuminuria status, duration of diabetes and Charlson Comorbidity Index including pre-existing cardiovascular diseases), diabetic complications (retinopathy and neuropathy), medication use within the 6 months before the index date (Oral anti-diabetic medications, insulin, anti-hypertensive medications, anticoagulants, antiplatelets, lipid-lowering agents and NSAIDs), and index year.

Fig. S3. Restricted cubic splines models for secondary outcomes with adjustment for all baseline covariates.


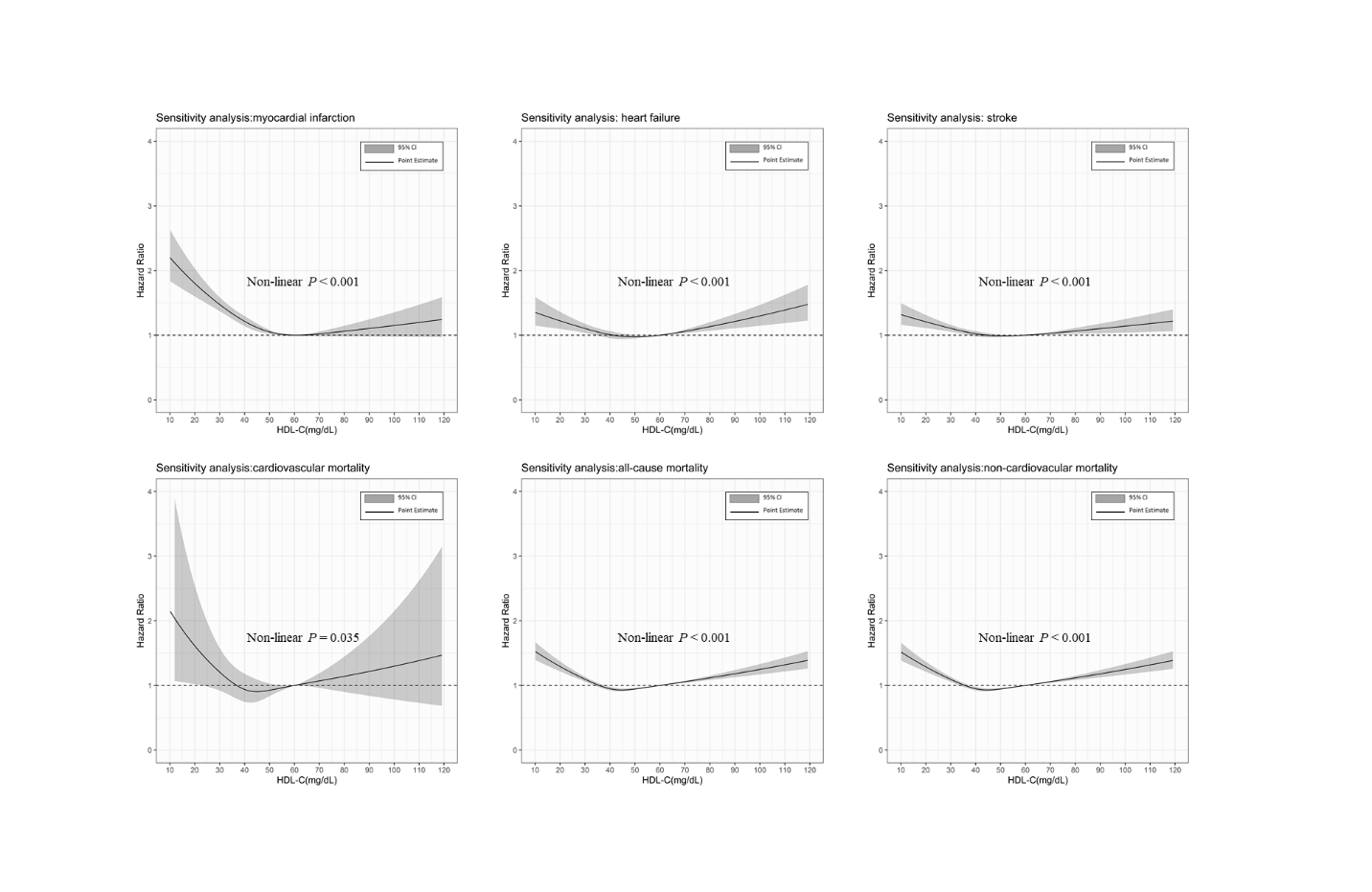


HDL-C = High-density lipoprotein cholesterol; CI = Confidence interval; Association between continuous HDL-C levels and incident secondary outcomes estimated by restricted cubic splines models with adjustment for all baseline covariates. The reference point was set at the HDL-C level of 60 mg/dL; Hazard ratio is calculated after adjusting for covariates, including sociodemographic characteristics (age, sex, smoking status, and alcohol status), clinical and laboratory parameters (BMI, HbA1c, fasting glucose, systolic blood pressure, diastolic blood pressure, LDL-C, triglyceride, eGFR, albuminuria status, duration of diabetes and Charlson Comorbidity Index including pre-existing cardiovascular diseases), diabetic complications (retinopathy and neuropathy), medication use within the 6 months before the index date (Oral anti-diabetic medications, insulin, anti-hypertensive medications, anticoagulants, antiplatelets, lipid-lowering agents and NSAIDs), and index year.

Fig. S4. Restricted cubic splines models for incident MACEs among individuals with more than 1-year follow-up.


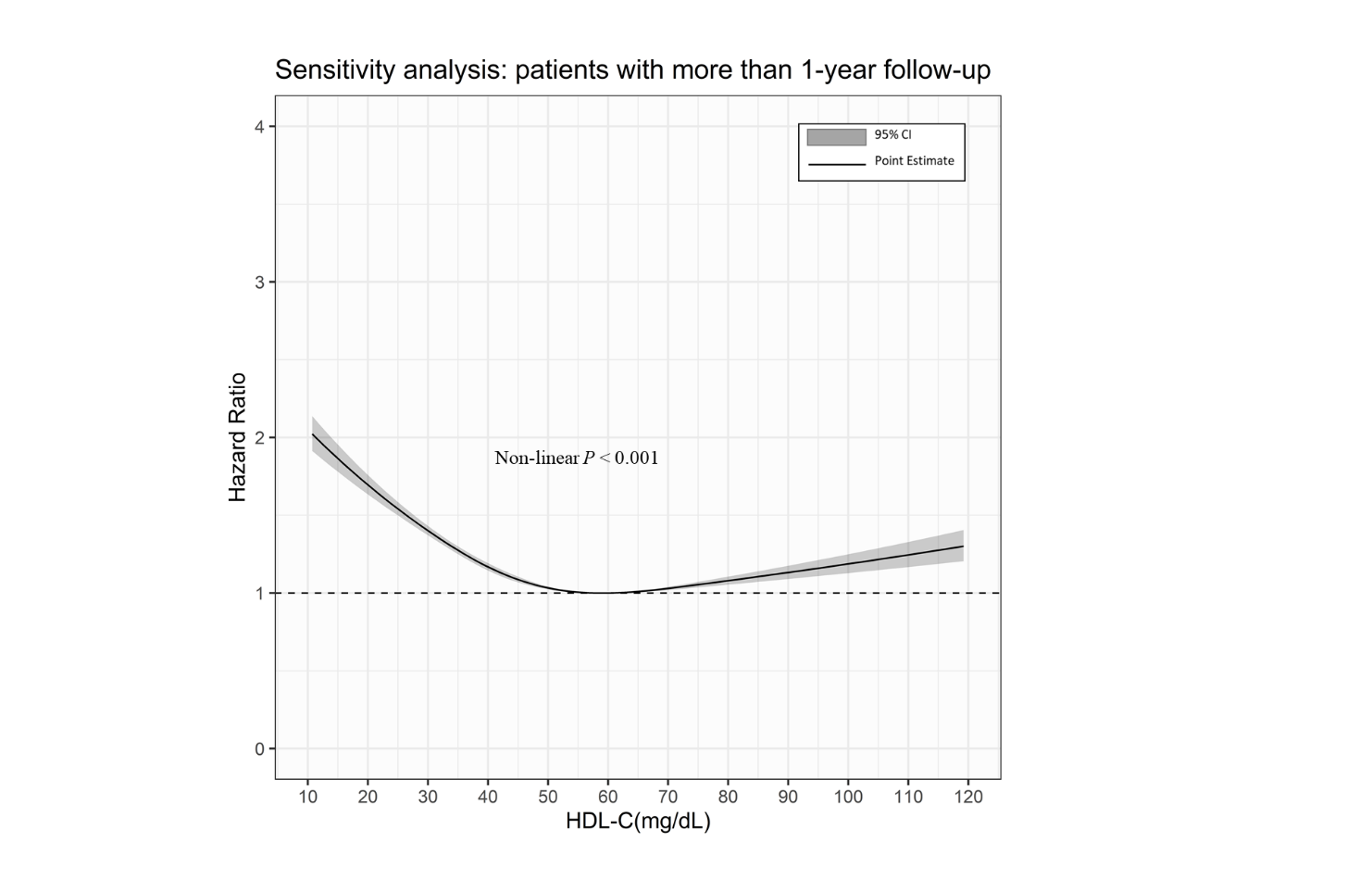


MACEs = Major adverse cardiovascular events; HDL-C = High-density lipoprotein cholesterol; CI = Confidence interval; The reference point was set at the HDL-C level of 60 mg/dL.

Fig. S5. Restricted cubic splines models for incident MACEs among individuals without any pre-existing cardiovascular diseases and procedures.


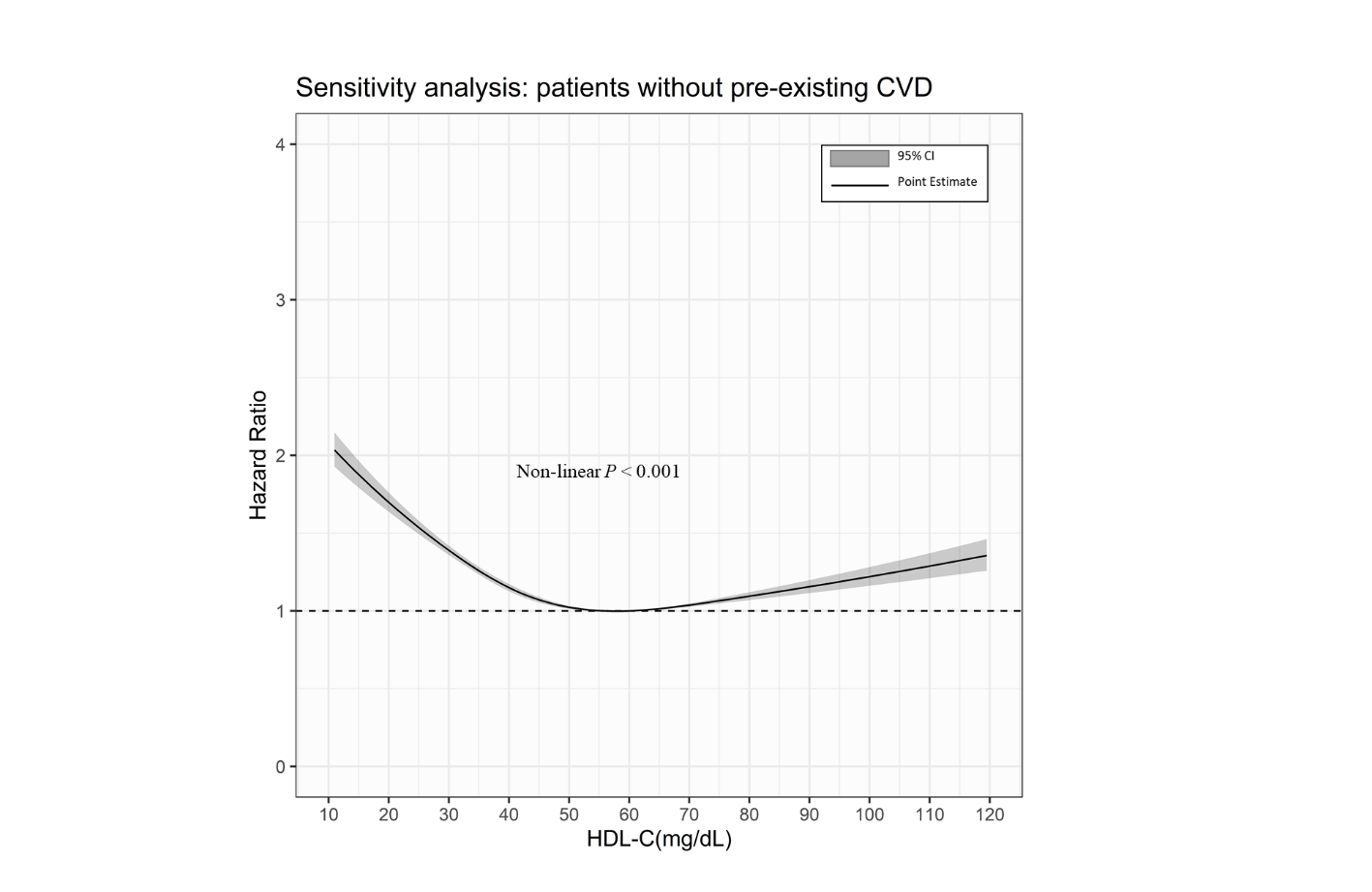


MACEs = Major adverse cardiovascular events; CVD = Cardiovascular diseases; HDL-C = High-density lipoprotein cholesterol; CI = Confidence interval; The reference point was set at the HDL-C level of 60 mg/dL.

Fig. S6. Restricted cubic splines models for incident 3-point MACEs among individuals without any pre-existing cardiovascular diseases and procedures.


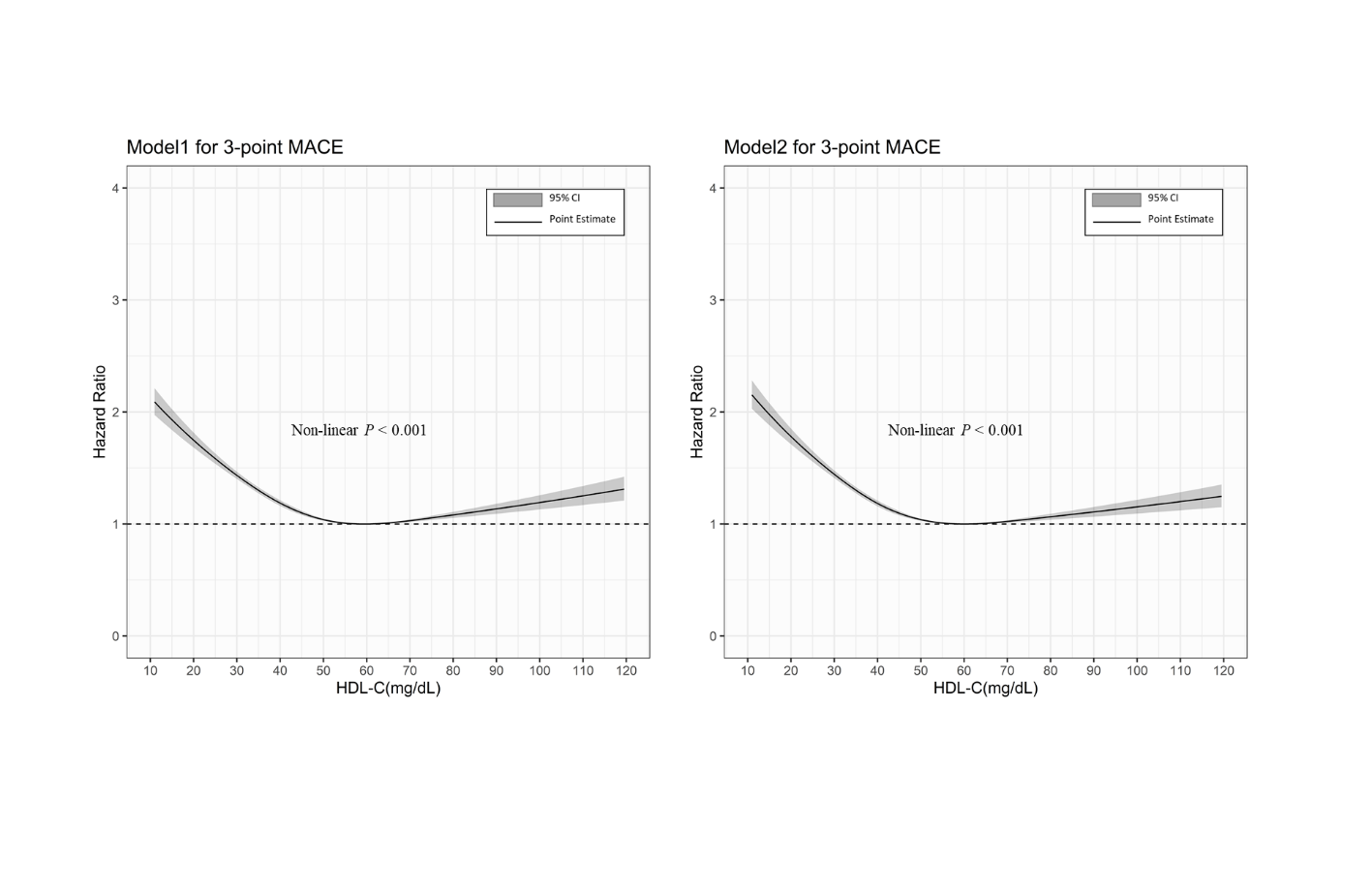


(b)

(a)

MACEs = Major adverse cardiovascular events; HDL-C = High-density lipoprotein cholesterol; CI = Confidence interval; The reference point was set at the HDL-C level of 60 mg/dL.

1. Model 1 without adjustment (Model 1); (b) Model 2 with adjustment for age, sex, and index year (Model 2)

Table S1. List of disease diagnosis codes, procedure codes, and laboratory criteria for each clinical diagnosis.

| Disease diagnosis | ICD-9-CM diagnosis code | ICD-9-CM procedure code | ICD-10-CM code | ICPC-2 | Laboratory criteria |
| --- | --- | --- | --- | --- | --- |
| Type 2 diabetes | 250.0[02] 250.1[02] 250.2[02] 250.3[02] 250.4[02] 250.5[02] 250.6[02] 250.7[02] 250.8[02] 250.9[02] |  |  | T90 |  |
| End-stage kidney disease | 585.5 585.6 586 | 39.95 54.98 55.6 |  |  | eGFR <15mL/min/1.73m2 |
| Dialysis | 585.6 V45.11 V56.0 | 39.95 54.98 |  |  |  |
| Kidney transplant | V42.0 996.81 | 55.61 55.69 |  |  |  |
| Cardiovascular disease | 410-414.9 428.x 430-438.99 | 36.03 36.04 36.06 36.07 36.09 36.1 36.10 36.11 36.12 36.13 36.14 36.15 36.16 36.17 36.19 36.2 36.31 36.32 36.33 36.34 36.39 36.91 36.99 |  | K74 K75 K76 K77 K89 K90 K91 |  |
| Major adverse cardiovascular events |  |  |  |  |  |
| Myocardial infarction | 410.x |  |  | K75 |  |
| Stroke | 430-438.99 |  |  | K89 K90 K91 |  |
| Heart Failure | 428.x |  |  | K77 |  |
| Cardiovascular mortality |  |  | I00-99 |  |  |
| Pre-existing cardiovascular diseases | 411-414.99 | 36.03 36.04 36.06 36.07 36.09 36.1 36.10 36.11 36.12 36.13 36.14 36.15 36.16 36.17 36.19 36.2 36.31 36.32 36.33 36.34 36.39 36.91 36.99 |  | K74 K76 |  |
| Diabetic retinopathy | 362.01 362.03-362.06 |  |  | F83 |  |
| Diabetic neuropathy | 249.6x 250.6x 337.1x 355.x 357.2 |  |  | N94 |  |

ICD-9-CM = International Classification of Diseases, 9th Revision, Clinical Modification; ICD-10-CM = International Classification of Diseases, 10th Revision, Clinical Modification; ICPC-2 = International Classification of Primary Care, Second Edition; eGFR = Estimated glomerular filtration rate.

Table S2. List of drug items and identify codes.

| **Drug** | **Items** | **BNF** |
| --- | --- | --- |
| **Anti-diabetic medications** | | |
| Insulin |  | 6.1.1 |
| Oral anti-diabetic medications | | 6.1.2 |
| SGLT2i | Canagliflozin, Dapagliflozin, Empagliflozin, Ertugliflozin |  |
| GLP1rA | Dulaglutide, Exenatide, Liraglutide, Lixisenatide |  |
| Metformin | Metformin HCL, Avandamet, Glucovance, Janumet, Kombiglyze XR, Xigduo XR |  |
| Sulfonylurea | Chlorpropamide, Glibenclamide, Gliclazide, Glimepiride, Glipizide, Glucovance, Tolbutamide |  |
| Thiazolidinedione | Pioglitazone (HCL), rosiglitazone (Maleate), Actosmet, Avandamet |  |
| DPP4i | Alogliptin (Benzoate), Galvus Met, Janumet, Kombiglyze XR, Linagliptin, Omarigliptin, Oseni, Saxagliptin (HCL), Sitagliptin (Phosphate), Trajenta Duo, Vildagliptin |  |
| **Anti-hypertensive medications** | |  |
| ACEI/ARB | Captopril, Cilazapril, Enalapril, Fosinopril, Lisinopril, Perindopril, Quinapril, Ramipril Azilsartan, Azoren, Candesartan, Exforge (Valsartan & Amlodipine), Irbesartan, Losartan, Olmesartan, Sparsentan/Irbesartan, Telmisartan, Telmisartan (Micardis Plus), Valsartan, Valsartan (Co-Diovan) | 2.5.5.1; 2.5.5.2 |
| Beta blockers | Acebutolol, Atenolol, Bisoprolol, Carvedilol, Celiprolol, Esmolol, Labetalol, Lodoz, Metoprolol, Nadolol, Nebivolol, Pindolol, Propranolol, Sotalol | 2.4 |
| Calcium channel blockers | Amlodipine, Azoren (Olmesartan Medoxomil & Amlodipine), Caduet (Amlodipine & Atorvastatin), Diltiazem, Exforge (Valsartan & Amlodipine), Felodipine, Gabapentin, Isradipine, Lacidipine, Lercanidipine, Nicardipine, Nifedipine, Nimodipine, Nitrendipine, Pregabalin, Verapamil | 2.6.2 |
| Diuretics | Amiloride, Bumetanide, Chlorthalidone, Co-Diovan (Valsartan & Hydrochlorothiazide), Dyazide (Triamterene & Hydrochlorothiazide), Eplerenone, Furosemide, Hydrochlorothiazide, Indapamide, Irbesartan & Hydrochlorothiazide, Lodoz (Bisoprolol & Hydrochlorothiazide), Losartan K & Hydrochlorothiazide, Metolazone, Micardis (Telmisartan & Hydrochlorothiazide), Moduretic (Amiloride & Hydrochlorothiazide), Spironolactone | 2.2 |
| Others anti-hypertensive drugs | Aliskiren, Clonidine, Hydralazine, Methyldopa, Minoxidil, Prazosin, Reserpine |  |
| **Anticoagulants** | Apixaban, Argatroban, AVE5026/Placebo, Dabigatran, Dalteparin sodium, Defibrotide, Edoxaban, Enoxaparin sodium prefilled syringe, Epoprostenol, Fondaparinux sodium prefilled syringe, Heparin calcium (Monoparin-CA), Heparin saline, Heparinised saline, Nadroparin calcium, Rivaroxaban, Tinzaparin sodium, Warfarin sodium | 2.8.1; 2.8.2 |
| **Antiplatelet** | Abciximab, Aggrenox (or equiv), Aspirin, Clopidogrel (hydrogen sulphate), Coplavix (or equiv), Dipyridamole, Eptifibatide, Persantin Plus (or equiv), Prasugrel (HCL), Ticagrelor, Ticlopidine HCL. | 2.9 |
| **Lipid-lowering agents** | | 2.12 |
| Statins | Atorvastatin, Caduet (Amlodipine & Atorvastatin), Fluvastatin, Lovastatin, Pravastatin, Rosuvastatin, Simvastatin, Vytorin (ezetimibe & simvastatin) |  |
| Fibrates | Bezafibrate, Clofibrate, Fenofibrate, Gemfibrozil |  |
| Ezetimibe | Ezetimibe |  |
| Other lipid-lowering agents | Acipimox, Alirocumab, Alpha tocopheryl nicotinate (DL) (VIT E), Benfluorex HCL, Bezafibrate SR, Cholestyramine, Clofibrate, Evolocumab (Repatha), Ezetimibe, Fenofibrate, Gemfibrozil, Maxepa, Nicotinic acid extended release, Nicotinic acid prolonged release, Nicotinic acid, Probucol, Tredaptive |  |
| **NSAIDs** | Celecoxib, Diclofenac Potassium, Diclofenac Sodium, Diflunisal, Etodolac, Etoricoxib, Flurbiprofen, Glucosamine Sulphate, Ibuprofen, Indomethacin, Ketoprofen, Lonazolac Calcium, Mefenamic Acid, Meloxicam, Nabumetone, Naproxen, Neurofenac (or equiv), Piroxicam, Proglumetacin Dimaleate (Protacin), Sulindac, Tenoxicam | 10.1.1 |

DPP4i = Dipeptidyl peptidase 4 inhibitors; SGLT2i = Sodium-glucose cotransporter 2 inhibitors; GLP1rA = Glucagon-like peptide 1 receptor agonists; ACEI/ARB = Angiotensin-converting enzyme inhibitors / Angiotensin receptor blockers; NSAIDs = Non-steroidal anti-inflammatory drugs; BNF = British National Formulary.

Table S3. Data completion rate.

|  | **HDL-C levels^a^** | | | |
| --- | --- | --- | --- | --- |
|  | **All individuals  (N=596,943)** | **Low HDL-C  (N=168,931)** | **Medium HDL-C  (N=412,863)** | **High HDL-C  (N=15,149)** |
|  | **Factors, % (n)** | **Factors, % (n)** | **Factors, % (n)** | **Factors, % (n)** |
| **Socio-demographics** | | | | |
| Sex | 100.0% (596,943) | 100.0% (168,931) | 100.0% (412,863) | 100.0% (15,149) |
| Age | 100.0% (596,943) | 100.0% (168,931) | 100.0% (412,863) | 100.0% (15,149) |
| Smoking status | 93.0% (555,396) | 91.7% (154,892) | 93.6% (386,388) | 93.2% (14,116) |
| Alcohol status | 81.7% (487,422) | 80.4% (135,761) | 82.3% (339,610) | 79.5% (12,051) |
| **Clinical and laboratory parameters** | | | | |
| Body mass index | 74.1% (442,160) | 69.1% (116,776) | 76.1% (314,130) | 74.3% (11,254) |
| HbA1c | 99.2% (591,937) | 99.3% (167,740) | 99.1% (409,302) | 98.3% (14,895) |
| Fasting glucose | 97.8% (583,516) | 97.9% (165,396) | 97.7% (403,330) | 97.6% (14,790) |
| Systolic blood pressure | 86.8% (518,153) | 83.7% (141,378) | 88.1% (363,573) | 87.1% (13,202) |
| Diastolic blood pressure | 86.8% (518,126) | 83.7% (141,366) | 88.1% (363,558) | 87.1% (13,202) |
| LDL-C | 99.9% (596,057) | 99.6% (168,234) | 100.0% (412,679) | 100.0% (15,144) |
| Triglyceride | 100.0% (596,701) | 99.9% (168,838) | 100.0% (412,722) | 99.9% (15,141) |
| eGFR | 99.9% (596,633) | 100.0% (168,866) | 99.9% (412,624) | 100.0% (15,143) |
| UACR | 76.4% (455,990) | 74.5% (125,934) | 77.2% (318,895) | 73.7% (11,161) |
| Duration of diabetes | 100.0% (596,943) | 100.0% (168,931) | 100.0% (412,863) | 100.0% (15,149) |
| CCI | 100.0% (596,943) | 100.0% (168,931) | 100.0% (412,863) | 100.0% (15,149) |
| Pre-existing cardiovascular diseases | 100.0% (596,943) | 100.0% (168,931) | 100.0% (412,863) | 100.0% (15,149) |
| Diabetic complications |  |  |  |  |
| Retinopathy | 100.0% (596,943) | 100.0% (168,931) | 100.0% (412,863) | 100.0% (15,149) |
| Neuropathy | 100.0% (596,943) | 100.0% (168,931) | 100.0% (412,863) | 100.0% (15,149) |
| **Use of medication (6 months prior to baseline)** | | | | |
| Anti-diabetic medications | 100.0% (596,943) | 100.0% (168,931) | 100.0% (412,863) | 100.0% (15,149) |
| Oral anti-diabetic medications | 100.0% (596,943) | 100.0% (168,931) | 100.0% (412,863) | 100.0% (15,149) |
| Metformin | 100.0% (596,943) | 100.0% (168,931) | 100.0% (412,863) | 100.0% (15,149) |
| Sulfonylurea | 100.0% (596,943) | 100.0% (168,931) | 100.0% (412,863) | 100.0% (15,149) |
| Thiazolidinedione | 100.0% (596,943) | 100.0% (168,931) | 100.0% (412,863) | 100.0% (15,149) |
| SGLT2i | 100.0% (596,943) | 100.0% (168,931) | 100.0% (412,863) | 100.0% (15,149) |
| GLP1rA | 100.0% (596,943) | 100.0% (168,931) | 100.0% (412,863) | 100.0% (15,149) |
| DPP4i | 100.0% (596,943) | 100.0% (168,931) | 100.0% (412,863) | 100.0% (15,149) |
| Alpha-glucosidase inhibitors | 100.0% (596,943) | 100.0% (168,931) | 100.0% (412,863) | 100.0% (15,149) |
| Insulin | 100.0% (596,943) | 100.0% (168,931) | 100.0% (412,863) | 100.0% (15,149) |
| Anti-hypertensive medications | 100.0% (596,943) | 100.0% (168,931) | 100.0% (412,863) | 100.0% (15,149) |
| ACEI/ARB | 100.0% (596,943) | 100.0% (168,931) | 100.0% (412,863) | 100.0% (15,149) |
| Beta-blockers | 100.0% (596,943) | 100.0% (168,931) | 100.0% (412,863) | 100.0% (15,149) |
| Calcium-channel blockers | 100.0% (596,943) | 100.0% (168,931) | 100.0% (412,863) | 100.0% (15,149) |
| Diuretics | 100.0% (596,943) | 100.0% (168,931) | 100.0% (412,863) | 100.0% (15,149) |
| Other anti-hypertensive medications | 100.0% (596,943) | 100.0% (168,931) | 100.0% (412,863) | 100.0% (15,149) |
| Anticoagulants | 100.0% (596,943) | 100.0% (168,931) | 100.0% (412,863) | 100.0% (15,149) |
| Antiplatelets | 100.0% (596,943) | 100.0% (168,931) | 100.0% (412,863) | 100.0% (15,149) |
| Lipid-lowering agents | 100.0% (596,943) | 100.0% (168,931) | 100.0% (412,863) | 100.0% (15,149) |
| Statins | 100.0% (596,943) | 100.0% (168,931) | 100.0% (412,863) | 100.0% (15,149) |
| Fibrates | 100.0% (596,943) | 100.0% (168,931) | 100.0% (412,863) | 100.0% (15,149) |
| Ezetimibe | 100.0% (596,943) | 100.0% (168,931) | 100.0% (412,863) | 100.0% (15,149) |
| Other lipid-lowering agents | 100.0% (596,943) | 100.0% (168,931) | 100.0% (412,863) | 100.0% (15,149) |
| NSAIDs | 100.0% (596,943) | 100.0% (168,931) | 100.0% (412,863) | 100.0% (15,149) |

HbA1c = Haemoglobin A1c; LDL-C = Low-density lipoprotein cholesterol; HDL-C = High-density lipoprotein cholesterol; eGFR = Estimated glomerular filtration rate; UACR = Urine Albumin-Creatinine Ratio; CCI = Charlson Comorbidity Index; DPP4i = Dipeptidyl peptidase 4 inhibitors; SGLT2i = Sodium-glucose cotransporter 2 inhibitors; GLP1rA = Glucagon-like peptide 1 receptor agonists; ACEI/ARB = Angiotensin-converting enzyme inhibitors / Angiotensin receptor blockers; NSAIDs = Non-steroidal anti-inflammatory drugs.

^a^HDL-C groups were classified by HDL-C levels: low HDL-C (≤40 mg/dL), medium HDL-C (>40 and ≤80 mg/dL) and high HDL-C (>80 mg/dL).

Table S4. Hazard ratios and crude incidence rate of secondary outcomes for low HDL-C and high HDL-C groups compared with medium HDL-C group.

|  |  |  |  |  | Model1^e^ | | |  | Model2^f^ | | |
| --- | --- | --- | --- | --- | --- | --- | --- | --- | --- | --- | --- |
| HDL-C levels^a^ | Event | Crude incidence rate^b^ | 95% CI^c^ | Person-years | HR^d^ | 95% CI | P-Value |  | HR | 95% CI | P-Value |
| Stroke | | | | | | | | | | | |
| Low HDL-C | 9,579 | 1.28 | (1.25, 1.30) | 749,615 | 1.086 | (1.058, 1.114) | <0.001 |  | 1.134 | (1.105, 1.164) | <0.001 |
| Medium HDL-C | 13,887 | 1.17 | (1.16, 1.19) | 1,182,009 | Ref | | |  | Ref | | |
| High HDL-C | 409 | 1.43 | (1.30, 1.58) | 28,543 | 1.221 | (1.107, 1.348) | <0.001 |  | 1.133 | (1.027, 1.250) | 0.013 |
| Heart Failure | | | | | | | | | | | |
| Low HDL-C | 6,469 | 0.84 | (0.82, 0.87) | 765,920 | 1.282 | (1.241, 1.325) | <0.001 |  | 1.310 | (1.268, 1.354) | <0.001 |
| Medium HDL-C | 7,915 | 0.66 | (0.64, 0.67) | 1,208,185 | Ref | | |  | Ref | | |
| High HDL-C | 236 | 0.81 | (0.71, 0.92) | 29,230 | 1.239 | (1.089, 1.410) | 0.001 |  | 1.155 | (1.015, 1.315) | 0.029 |
| Myocardial infarction | | | | | | | | | | | |
| Low HDL-C | 5,666 | 0.74 | (0.72, 0.76) | 768,956 | 1.421 | (1.370, 1.473) | <0.001 |  | 1.441 | (1.390, 1.494) | <0.001 |
| Medium HDL-C | 6,250 | 0.51 | (0.50, 0.53) | 1,214,533 | Ref | | |  | Ref | | |
| High HDL-C | 163 | 0.55 | (0.47, 0.65) | 29,462 | 1.087 | (0.930, 1.270) | 0.293 |  | 1.037 | (0.888, 1.212) | 0.645 |
| Cardiovascular mortality | | | | | | | | | | | |
| Low HDL-C | 319 | 0.04 | (0.04, 0.05) | 784,690 | 1.134 | (0.981, 1.311) | 0.089 |  | 1.193 | (1.032, 1.380) | 0.017 |
| Medium HDL-C | 433 | 0.04 | (0.03, 0.04) | 1,230,438 | Ref | | |  | Ref | | |
| High HDL-C | 11 | 0.04 | (0.02, 0.07) | 29,767 | 1.064 | (0.585, 1.936) | 0.839 |  | 0.978 | (0.537, 1.779) | 0.941 |
| Non-cardiovascular mortality | | | | | | | | | | | |
| Low HDL-C | 20,978 | 2.67 | (2.64, 2.71) | 784,690 | 1.047 | (1.029, 1.066) | <0.001 |  | 1.127 | (1.107, 1.147) | <0.001 |
| Medium HDL-C | 30,699 | 2.49 | (2.47, 2.52) | 1,230,438 | Ref | | |  | Ref | | |
| High HDL-C | 1,275 | 4.28 | (4.05, 4.52) | 29,767 | 1.754 | (1.658, 1.855) | <0.001 |  | 1.608 | (1.521, 1.701) | <0.001 |
| All-cause mortality | | | | | | | | | | | |
| Low HDL-C | 21,297 | 2.714 | (2.68, 2.75) | 784,690 | 1.048 | (1.030, 1.067) | <0.001 |  | 1.127 | (1.108, 1.147) | <0.001 |
| Medium HDL-C | 31,132 | 2.530 | (2.50, 2.56) | 1,230,438 |  |  |  |  |  |  |  |
| High HDL-C | 1,286 | 4.320 | (4.09, 4.56) | 29,767 | 1.744 | (1.649, 1.844) | <0.001 |  | 1.600 | (1.513, 1.691) | <0.001 |

MACEs = Major adverse cardiovascular events; HDL-C = High-density lipoprotein cholesterol; HR = Hazard ratio; CI = Confidence interval.

^a^HDL-C groups were classified by HDL-C levels: low HDL-C (≤40 mg/dL), medium HDL-C (>40 and ≤80 mg/dL) and high HDL-C (>80 mg/dL).

^b^The unit of crude incidence rate: events per 100 person-years.

^c^The 95% CIs of the crude incidence rates were generated according to the Poisson distribution.

^d^HR >1 (or <1) indicates low/high HDL-C group had higher (lower) risk of MACE outcomes compared to medium HDL-C groups.

^e^Model1: model without adjustment.

^f^Model2: model adjusted for age, sex, and index year.

Table S5. Baseline Characteristics for Individuals Without Missing Covariates and Stratified by Different HDL-C Categories.

|  | HDL-C levels^a^ | | | |  |
| --- | --- | --- | --- | --- | --- |
|  | Overall (N = 328,410) | Low HDL-C (N = 86,373) | Medium HDL-C (N = 234,172) | High HDL-C (N = 7,865) | P-value |
| **Socio-demographics** |  |  |  |  |  |
| Sex, No. (%) |  |  |  |  |  |
| Male | 161,498 (49.18) | 57,383 (66.44) | 101,752 (43.45) | 2,363 (30.04) | <0.001 |
| Female | 166,912 (50.82) | 28,990 (33.56) | 132,420 (56.55) | 5,502 (69.96) |  |
| Age, mean (SD), y | 61.8 (11.5) | 60.1 (12.1) | 62.3 (11.2) | 65.6 (11.1) | <0.001 |
| Smoking status, No. (%) |  |  |  |  |  |
| Non-smoker | 235,896 (71.83) | 52,131 (60.36) | 177,304 (75.72) | 6,461 (82.15) | <0.001 |
| Ex-smoker | 49,013 (14.92) | 15,888 (18.39) | 32,264 (13.78) | 861 (10.95) |  |
| Current smoker | 43,501 (13.25) | 18,354 (21.25) | 24,604 (10.51) | 543 (6.90) |  |
| Alcohol status, No. (%) |  |  |  |  |  |
| Non-drinker | 239,018 (72.78) | 59,184 (68.52) | 173,785 (74.21) | 6,049 (76.91) | <0.001 |
| Ex-drinker | 22,209 (6.76) | 7,446 (8.62) | 14,362 (6.13) | 401 (5.10) |  |
| Current drinker | 67,183 (20.46) | 19,743 (22.86) | 46,025 (19.65) | 1,415 (17.99) |  |
| **Clinical and laboratory parameters** |  |  |  |  |  |
| Body mass index, mean (SD), kg/m2 | 26.1 (4.2) | 26.9 (4.2) | 25.9 (4.2) | 23.3 (4.2) | <0.001 |
| HbA1c, mean (SD) |  |  |  |  |  |
| % | 7.6 (1.8) | 7.8 (1.9) | 7.5 (1.7) | 7.3 (1.8) | <0.001 |
| mmol/mol | 60.0 (19.7) | 62.0 (20.8) | 58.0 (18.6) | 56.0 (19.7) |  |
| Fasting glucose, mean (SD), mmol/L | 7.9 (2.7) | 8.2 (2.9) | 7.8 (2.6) | 7.6 (2.7) | <0.001 |
| Blood pressure, mean (SD), mm Hg |  |  |  |  |  |
| Systolic | 134.9 (17.1) | 135.1 (17.2) | 134.8 (17.0) | 134.8 (17.7) | 0.020 |
| Diastolic | 76.7 (10.7) | 78.0 (11.0) | 76.3 (10.5) | 73.6 (10.4) | <0.001 |
| LDL-C, mean (SD), mmol/L | 2.9 (0.9) | 2.8 (0.9) | 3.0 (0.9) | 2.7 (0.9) | <0.001 |
| Triglyceride, mean (SD), mmol/L | 1.7 (1.2) | 2.4 (1.7) | 1.5 (0.9) | 0.9 (0.4) | <0.001 |
| eGFR, mean (SD), mL/min/1.73m2 | 84.9 (20.0) | 84.2 (20.1) | 85.1 (20.0) | 83.8 (17.9) | <0.001 |
| Albuminuria status, No. (%) |  |  |  |  |  |
| UACR <3 mg/mmol | 237,218 (72.23) | 58,903 (68.20) | 172,382 (73.61) | 5,933 (75.44) | <0.001 |
| UACR 3-30 mg/mmol | 75,081 (22.86) | 22,051 (25.53) | 51,470 (21.98) | 1,560 (19.83) |  |
| UACR >30 mg/mmol | 16,111 (4.91) | 5,419 (6.27) | 10,320 (4.41) | 372 (4.73) |  |
| Duration of diabetes, mean (SD), y | 2.6 (4.2) | 2.6 (4.2) | 2.5 (4.2) | 3.0 (4.6) | <0.001 |
| CCI, mean (SD) | 4.1 (1.6) | 4.0 (1.6) | 4.1 (1.5) | 4.5 (1.5) | <0.001 |
| Pre-existing cardiovascular diseases, No. (%) | 13,489 (4.1) | 4,203 (4.9) | 9,072 (3.9) | 214 (2.7) | <0.001 |
| Diabetic complications, No. (%) |  |  |  |  |  |
| Retinopathy | 4,200 (1.28) | 1,116 (1.29) | 2,985 (1.27) | 99 (1.26) | 0.915 |
| Neuropathy | 1,050 (0.32) | 314 (0.36) | 708 (0.30) | 28 (0.36) | 0.021 |
| **Use of medication (6 months prior to baseline)** **, No. (%)** |  |  |  |  |  |
| Anti-diabetic medications | 243,704 (74.21) | 68,427 (79.22) | 170,024 (72.61) | 5,253 (66.79) | <0.001 |
| Oral anti-diabetic medications | 242,326 (73.79) | 67,979 (78.70) | 169,170 (72.24) | 5,177 (65.82) | <0.001 |
| Metformin | 222,085 (67.62) | 62,080 (71.87) | 155,407 (66.36) | 4,598 (58.46) | <0.001 |
| Sulfonylurea | 111,188 (33.86) | 34,424 (39.86) | 74,478 (31.80) | 2,286 (29.07) | <0.001 |
| Thiazolidinedione | 1,041 (0.32) | 327 (0.38) | 687 (0.29) | 27 (0.34) | <0.001 |
| SGLT2i | 271 (0.08) | 110 (0.13) | 153 (0.07) | 8 (0.10) | <0.001 |
| GLP1rA | 40 (0.01) | 14 (0.02) | 25 (0.01) | 1 (0.01) | 0.452 |
| DPP4i | 1,878 (0.57) | 708 (0.82) | 1,134 (0.48) | 36 (0.46) | <0.001 |
| Alpha-glucosidase inhibitors | 516 (0.16) | 180 (0.21) | 326 (0.14) | 10 (0.13) | <0.001 |
| Insulin | 13,306 (4.05) | 5,318 (6.16) | 7,634 (3.26) | 354 (4.50) | <0.001 |
| Anti-hypertensive medications | 226,910 (69.09) | 60,380 (69.91) | 161,393 (68.92) | 5,137 (65.31) | <0.001 |
| ACEI/ARB | 107,095 (32.61) | 30,881 (35.75) | 74,018 (31.61) | 2,196 (27.92) | <0.001 |
| Beta-blockers | 79,041 (24.07) | 24,375 (28.22) | 53,431 (22.82) | 1,235 (15.70) | <0.001 |
| Calcium-channel blockers | 157,356 (47.91) | 40,707 (47.13) | 112,913 (48.22) | 3,736 (47.50) | <0.001 |
| Diuretics | 29,999 (9.13) | 8,045 (9.31) | 21,231 (9.07) | 723 (9.19) | 0.095 |
| Other anti-hypertensive medications | 21,875 (6.66) | 6,715 (7.77) | 14,737 (6.29) | 423 (5.38) | <0.001 |
| Anticoagulants | 1,666 (0.51) | 667 (0.77) | 972 (0.42) | 27 (0.34) | <0.001 |
| Antiplatelets | 23,819 (7.25) | 7,546 (8.74) | 15,827 (6.76) | 446 (5.67) | <0.001 |
| Lipid-lowering agents | 109,249 (33.27) | 26,582 (30.78) | 80,441 (34.35) | 2,226 (28.30) | <0.001 |
| Statins | 101,339 (30.86) | 22,393 (25.93) | 76,782 (32.79) | 2,164 (27.51) | <0.001 |
| Fibrates | 9,465 (2.88) | 4,881 (5.65) | 4,511 (1.93) | 73 (0.93) | <0.001 |
| Ezetimibe | 280 (0.09) | 100 (0.12) | 176 (0.08) | 4 (0.05) | 0.001 |
| Other lipid-lowering agents | 80 (0.02) | 27 (0.03) | 50 (0.02) | 3 (0.04) | 0.205 |
| NSAIDs | 42,929 (13.07) | 11,761 (13.62) | 30,294 (12.94) | 874 (11.11) | <0.001 |

SD = standard deviation; HbA1c = Haemoglobin A1c; LDL-C = Low-density lipoprotein cholesterol; HDL-C = High-density lipoprotein cholesterol; eGFR = Estimated glomerular filtration rate; UACR = Urine Albumin-Creatinine Ratio; CCI = Charlson Comorbidity Index; DPP4i = Dipeptidyl peptidase 4 inhibitors; SGLT2i = Sodium-glucose cotransporter 2 inhibitors; GLP1rA = Glucagon-like peptide 1 receptor agonists; ACEI/ARB = Angiotensin-converting enzyme inhibitors / Angiotensin receptor blockers; NSAIDs = Non-steroidal anti-inflammatory drugs; ^a^ HDL-C groups were classified by HDL-C levels: low HDL-C (≤40 mg/dL), medium HDL-C (>40 and ≤80 mg/dL) and high HDL-C (>80 mg/dL).

Table S6. Hazard Ratios and Crude Incidence Rate of MACEs among Individuals Without Missing Covariates.

| **HDL-C levels** | **N** | **Event** | **Crude incidence rate^a^** | **95% CI†** | **Person-years** | **HR‡** | **95% CI†** | **P-value** |
| --- | --- | --- | --- | --- | --- | --- | --- | --- |
| Low HDL-C | 86,373 | 10,402 | 1.87 | (1.84, 1.91) | 555,198 | 1.075 | (1.048, 1.103) | <0.001 |
| Medium HDL-C | 234,172 | 23,491 | 1.56 | (1.54, 1.58) | 1,504,805 | Ref | | |
| High HDL-C | 7,865 | 917 | 1.83 | (1.71,1.95) | 50,121 | 1.162 | (1.087, 1.242) | <0.001 |

MACE = Major adverse cardiovascular events; HDL-C = High-density lipoprotein cholesterol; HR = Hazard ratio; CI = Confidence interval. ^a^ HDL-C groups were classified by HDL-C levels: low HDL-C (≤40 mg/dL), medium HDL-C (>40 and ≤80 mg/dL) and high HDL-C (>80 mg/dL). ^b^ The unit of crude incidence rate: events per 100 person-years; The 95% CIs of the crude incidence rates were generated according to the Poisson distribution. ^c^ Hazard ratio is calculated after adjusting for covariates, including sociodemographic characteristics (age, sex, smoking status, and alcohol status), clinical and laboratory parameters (BMI, HbA1c, fasting glucose, systolic blood pressure, diastolic blood pressure, LDL-C, triglyceride, eGFR, albuminuria status, duration of diabetes, and Charlson Comorbidity Index including pre-existing cardiovascular diseases), diabetic complications (retinopathy and neuropathy), medication use within the 6 months before the index date (oral anti-diabetic medications, insulin, anti-hypertensive medications, anticoagulants, antiplatelets, lipid-lowering agents and NSAIDs), and index year. HR >1 (or <1) indicates low/high HDL-C group had higher (lower) risk of MACE outcomes compared to medium HDL-C groups

Table S7. Hazard ratios and crude incidence rate of MACEs for low HDL-C and high HDL-C groups compared with medium HDL-C group for male.

|  |  |  |  |  | Model1^e^ | | |  | Model2^f^ | | |
| --- | --- | --- | --- | --- | --- | --- | --- | --- | --- | --- | --- |
| HDL-C levels^a^ | Event | Crude incidence rate^b^ | 95% CI^c^ | Person-years | HR^d^ | 95% CI | P-value |  | HR | 95% CI | P-value |
| Low HDL-C | 17,434 | 2.41 | (2.37, 2.44) | 724,233 | 1.191 | (1.168, 1.215) | <0.001 |  | 1.237 | (1.212, 1.261) | <0.001 |
| Medium HDL-C | 23,249 | 2.02 | (1.99, 2.04) | 1,153,393 | Ref | | |  | Ref | | |
| High HDL-C | 680 | 2.44 | (2.26, 2.63) | 27,879 | 1.213 | (1.124, 1.309) | <0.001 |  | 1.128 | (1.045, 1.217) | 0.002 |

MACEs = Major adverse cardiovascular events; HDL-C = High-density lipoprotein cholesterol; HR = Hazard ratio; CI = Confidence interval.

^a^HDL-C groups were classified by HDL-C levels: low HDL-C (≤40 mg/dL), medium HDL-C (>40 and ≤80 mg/dL) and high HDL-C (>80 mg/dL).

^b^The unit of crude incidence rate: events per 100 person-years.

^c^The 95% CIs of the crude incidence rates were generated according to the Poisson distribution.

^d^HR >1 (or <1) indicates low/high HDL-C group had higher (lower) risk of MACE outcomes compared to medium HDL-C groups.

^e^Model1: model without adjustment.

^f^Model2: model adjusted for age, sex, and index year.

Table S8. Hazard ratios and crude incidence rate of MACEs for low HDL-C and high HDL-C groups compared with medium HDL-C group for female.

|  |  |  |  |  | Model1^e^ | | |  | Model2^f^ | | |
| --- | --- | --- | --- | --- | --- | --- | --- | --- | --- | --- | --- |
| HDL-C levels^a^ | Event | Crude incidence rate^b^ | 95% CI^c^ | Person-years | HR^d^ | 95% CI | P-value |  | HR | 95% CI | P-value |
| Low HDL-C | 22,083 | 2.18 | (2.15, 2.20) | 1,015,086 | 1.196 | (1.172, 1.220) | <0.001 |  | 1.183 | (1.160, 1.208) | <0.001 |
| Medium HDL-C | 16,789 | 1.81 | (1.78, 1.84) | 927,952 | Ref | | |  | Ref | | |
| High HDL-C | 1,357 | 1.97 | (1.86, 2.07) | 69,033 | 1.089 | (1.030, 1.151) | 0.003 |  | 1.079 | (1.021, 1.141) | 0.007 |

MACEs = Major adverse cardiovascular events; HDL-C = High-density lipoprotein cholesterol; HR = Hazard ratio; CI = Confidence interval.

^a^HDL-C groups were classified by HDL-C levels: low HDL-C (≤50 mg/dL), medium HDL-C (>50 and ≤80 mg/dL) and high HDL-C (>80 mg/dL).

^b^The unit of crude incidence rate: events per 100 person-years.

^c^The 95% CIs of the crude incidence rates were generated according to the Poisson distribution.

^d^HR >1 (or <1) indicates low/high HDL-C group had higher (lower) risk of MACE outcomes compared to medium HDL-C groups.

^e^Model1: model without adjustment.

^f^Model2: model adjusted for age, sex, and index year.

Table S9. Hazard ratios and crude incidence rate of 3-point MACEs for low HDL-C and high HDL-C groups compared with medium HDL-C group.

|  |  |  |  |  | Model1^e^ | | |  | Model2^f^ | | |
| --- | --- | --- | --- | --- | --- | --- | --- | --- | --- | --- | --- |
| HDL-C levels^a^ | Event | Crude incidence rate^b^ | 95% CI^c^ | Person-years | HR^d^ | 95% CI | P-value |  | HR | 95% CI | P-value |
| Low HDL-C | 21,391 | 1.89 | (1.87, 1.92) | 1,131,490 | 1.248 | (1.227, 1.268) | <0.001 |  | 1.242 | (1.221, 1.263) | <0.001 |
| Medium HDL-C | 41,534 | 1.51 | (1.50, 1.53) | 2,748,401 | Ref | | |  | Ref | | |
| High HDL-C | 1,590 | 1.62 | (1.54, 1.70) | 98,298 | 1.074 | (1.021, 1.129) | 0.005 |  | 1.063 | (1.011, 1.118) | 0.016 |

MACEs = Major adverse cardiovascular events; HDL-C = High-density lipoprotein cholesterol; HR = Hazard ratio; CI = Confidence interval.

^a^HDL-C groups were classified by HDL-C levels: low HDL-C (≤40 mg/dL), medium HDL-C (>40 and ≤80 mg/dL) and high HDL-C (>80 mg/dL).

^b^The unit of crude incidence rate: events per 100 person-years.

^c^The 95% CIs of the crude incidence rates were generated according to the Poisson distribution.

^d^HR >1 (or <1) indicates low/high HDL-C group had higher (lower) risk of MACE outcomes compared to medium HDL-C groups.

^e^Model1: model without adjustment.

^f^Model2: model adjusted for age, sex, and index year.
